# C/EBPβ regulates delta-secretase expression and mediates pathogenesis in mouse models of Alzheimer’s disease

**DOI:** 10.1038/s41467-018-04120-z

**Published:** 2018-05-03

**Authors:** Zhi-Hao Wang, Ke Gong, Xia Liu, Zhentao Zhang, Xiaoou Sun, Zheng Zachory Wei, Shan Ping Yu, Fredric P. Manfredsson, Ivette M. Sandoval, Peter F. Johnson, Jianping Jia, Jian-Zhi Wang, Keqiang Ye

**Affiliations:** 1 Department of Pathology and Laboratory Medicine, Emory University School of Medicine, Atlanta, GA 30322 USA; 2 Department of Pathophysiology, Key Laboratory of Ministry of Education of Neurological Diseases, Tongji Medical College, Huazhong University of Science and Technology, 430030 Wuhan, China; 3 Department of Anesthesiology, Emory University School of Medicine, Atlanta, GA 30322 USA; 4 Department of Translational Science & Molecular Medicine, Michigan State University, 333 Bostwick Ave NE, Grand Rapids, MI 49503 USA; 5 Mouse Cancer Genetics Program, Center for Cancer Research, National Cancer Institute, Frederick, MD 21702 USA; 6 Department of Neurology, Xuanwu Hospital of Capital Medical University, 100053 Beijing, China; 7 Translational Center for Stem Cell Research, Tongji Hospital, Tongji University School of Medicine, 200065 Shanghai, China

**Keywords:** Transcription, Alzheimer's disease

## Abstract

Delta-secretase cleaves both APP and Tau to mediate the formation of amyloid plaques and neurofibrillary tangle in Alzheimer’s disease (AD). However, how aging contributes to an increase in delta-secretase expression and AD pathologies remains unclear. Here we show that a CCAAT-enhancer-binding protein (C/EBPβ), an inflammation-regulated transcription factor, acts as a key age-dependent effector elevating both delta-secretase (AEP) and inflammatory cytokines expression in mediating pathogenesis in AD mouse models. We find that C/EBPβ regulates delta-secretase transcription and protein levels in an age-dependent manner. Overexpression of C/EBPβ in young 3xTg mice increases delta-secretase and accelerates the pathological features including cognitive dysfunctions, which is abolished by inactive AEP C189S. Conversely, depletion of C/EBPβ from old 3xTg or 5XFAD mice diminishes delta-secretase and reduces AD pathologies, leading to amelioration of cognitive impairment in these AD mouse models. Thus, our findings support that C/EBPβ plays a pivotal role in AD pathogenesis via increasing delta-secretase expression.

## Abstract (web summary)

Delta-secretase cleaves both APP and Tau, and contributes to Alzheimer’s disease-like pathology. Here the authors show that C/EBPβ, a regulator of inflammation, also regulates transcription of delta-secretase in an age-dependent manner and contributes to Alzheimer’s disease-like pathology in mouse models.

## Introduction

Alzheimer’s disease (AD) is characterized by the accumulation of Aβ peptide (Aβ) within the brain along with hyperphosphorylated and cleaved forms of the microtubule-associated protein Tau. The greatest known risk factor for AD is increasing age. However, the molecular mechanisms by which aging mediates the onset and progression of AD remain unclear. It is known that a metabolic dysfunction of amyloid β precursor protein (APP) and abnormal Tau protein phosphorylation lead to the formation of senile plaques and neurofibrillary tangles (NFT), respectively. These crucial events drive neurodegeneration and the clinical expression of dementia^1^. We recently reported that AEP (Legumain, LGMN), newly designated as delta-secretase, cleaves both APP and Tau in an age-dependent manner^2,3^. Delta-secretase expression levels and activity are escalated in aged mice and AD brains. Removal of delta-secretase from 5XFAD or Tau P301S mice ameliorates these pathological defects and rescues the cognitive functions in both animal models, suggesting that delta-secretase plays an essential role in AD onset and progression^2,3^. AEP is a lysosomal asparagine endopeptidase, and its activation is autocatalytic and requires sequential removal of N- and C-terminal peptides at different pH^4^. Previously, we reported that AEP cleaves SET that acts as a DNase inhibitor and inactivates SET, leading to DNA damage and neuronal cell death in a model of stroke^5^. In addition, SET also functions as a PP2A inhibitor. AEP-cleaved SET blocks the phosphatase activity of PP2A and results in Tau hyperphosphorylation and aggregation in AD^6,7^. Hence, delta-secretase plays a critical role in mediating AD pathogenesis.

In AD, one of the pathological hallmarks is chronic neuro-inflammation mediated by astrocytes and microglial cells. Accumulation and deposition of Aβ trigger the activation of microglia, which initiates an inflammatory response that, over time, becomes a chronic deleterious condition^8^. The transcription factors CCAAT/enhancer binding proteins (C/EBP) α, β, and δ are expressed in the brain and are involved in regulation of inflammatory genes in concert with nuclear factor κB (NF-κB). The C/EBP family of transcription factors comprises pleiotropic proteins involved in tissue-specific metabolic gene transcription, signal transduction activated by several cytokines, and in cell differentiation^9–14^. C/EBPs belong to the basic-leucine zipper DNA-binding protein family and have recently been implicated in CNS inflammation^15^. C/EBPs regulate the expression of genes critical to glia activation^16^, and C/EBP binding sites have been identified in the promoter regions of numerous cytokines and other pro-inflammatory genes^17^. Moreover, C/EBPs have been implicated in the inflammation observed in neurodegenerative diseases^16,18,19^ and brain injury^20^. In addition, C/EBPs also play roles in transcriptional programs underlying more complex brain functions, such as learning and memory^21^. Notably, C/EBPs are upregulated in AD^22,23^, and Aβ mediates C/EBP β and δ activation in glia cells^24^. Remarkably, C/EBP β −/− mice exhibit resistance to excitotoxicity-induced neuronal cell death^25^, indicating that this protein might regulate gene expression implicated in brain injury. Depletion of C/EBP β decreases ischemia (middle cerebral artery occlusion, MCAO)-induced brain damage and inflammation^26^. Interestingly, C/EBPs are also involved in aging and liver regeneration^27^. Noticeably, C/EBPβ and δ are increased in aged mouse brains, and mediate age-dependent response following brain injury^28^.

In the current study, we report that C/EBPβ regulates the expression of delta-secretase in an age-dependent manner. Overexpression of C/EBPβ escalates delta-secretase and neuro-inflammation, whereas deletion of C/EBPβ suppresses their expression levels. Overexpression of C/EBPβ in young 3xTg mice facilitates earlier onset of AD-like pathologies, leading to exacerbation of cognitive impairments via upregulating delta-secretase, neuro-inflammation and neuronal loss. By contrast, depletion of C/EBPβ from 5XFAD or old 3xTg mouse models significantly reduces these events, resulting in evident restoration of the cognitive functions.

## Results

### OGD increases delta-secretase expression via C/EBPβ

**Fig. 1:**
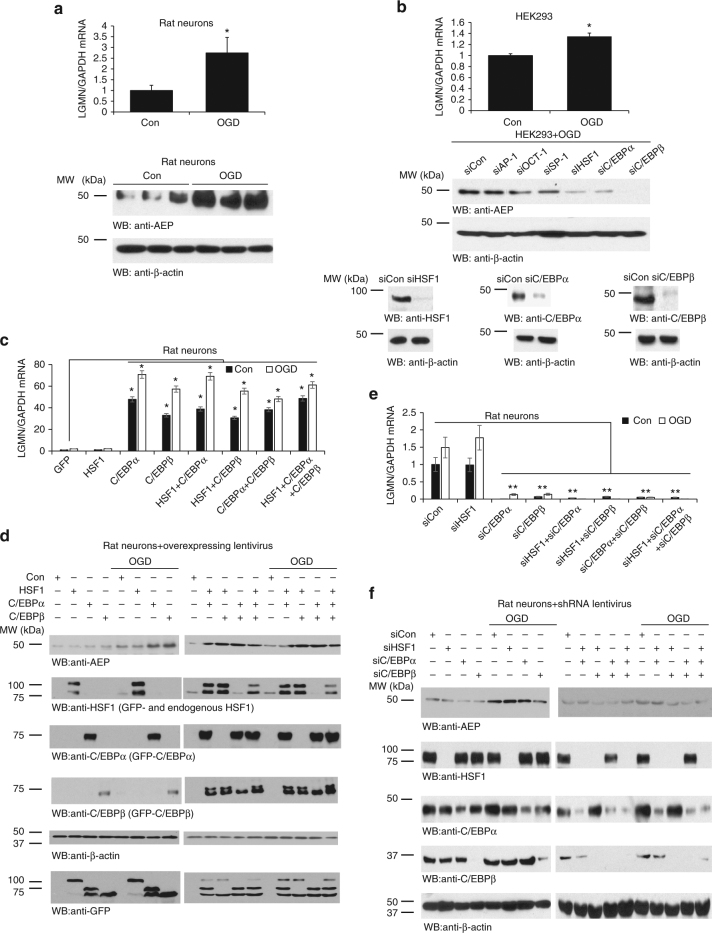
Oxygen glucose deprivation (OGD)/reperfusion promotes delta-secretase expression, and C/EBPs control delta-secretase expression in primary neurons. **a** OGD increases delta-secretase expression in neurons. Primary cultures of neurons from rat E17 embryos were seeded in a six-well plate. At DIV 7, neurons were treated with OGD for 2 h. After 24 h of recovery, neurons were collected for western blotting and real-time PCR analysis. At least three wells of neurons were tested in each experiment. *P < 0.05. **b** OGD increases delta-secretase expression in HEK293 cells. After 10 h of OGD treatment, HEK293 cells were recovered for 12 h; mRNAs were extracted and real-time PCR analysis. All real-time PCR experiments were repeated at least three times. β-actin was employed as a loading control. HEK293 cells were seeded and transfected with the various siRNAs. Forty-eight hours post transfection, all cells were given the OGD/recovery treatment. Cells lysates were analyzed by western blotting (middle panels). Knockdown of HSF, C/EBPα, and C/EBPβ was confirmed by western blotting (lower panels). **c**, **d** Overexpression of HSF1, C/EBPα, and C/EBPβ by lentivirus transduction of primary neurons increases delta-secretase expression. **e**, **f** Knock-down of these genes via lentivirus-mediated shRNA expression in primary neurons decreases delta-secretase expression. The primary cultures were treated by OGD 2 h/reperfusion for 24 h. Neurons were collected and lysed. The mRNA and protein levels of AEP were detected by real-time PCR (**c**, **e**) or western blotting analysis (**d**, **f**). HSF1, C/EBPα, and C/EBPβ’s expression levels were detected by western blotting as well (**d**,** f**). Western blot data in **a**, **b**, **d**, and **f** are representative of three independent experiments. Real-time PCR data in **a**–**c**, **e** represent mean ± s.e.m. of three independent experiments (**a**–**c**, **e**: *P < 0.05, Student’s *t*-test)

Delta-secretase (AEP, gene name: *LGMN*) mRNA and protein expression levels are increased in the peri-infarct area of rats after transient MCAO, and levels are upregulated in microglial cells and reactive astrocytes in the ischemic brain^29^. Oxygen-glucose deprivation (OGD) is a frequently utilized in vitro cellular model for ischemia and acute aging^30^. To explore whether OGD stimulation might regulate delta-secretase expression in neurons, we treated rat primary cortical cultures with OGD for 2 h, followed by 24 h reperfusion, and observed evident escalation of LGMN mRNA and protein levels (Fig. 1a). HEK293 cells express high levels of AEP^5^ and are amenable to genetic manipulation. Accordingly, we treated HEK293 with OGD and found that OGD also effectively increased mRNA levels of delta-secretase (Fig. 1b, top panel) demonstrating that OGD strongly increases delta-secretase expression in different cell types. These OGD/reperfusion treatment conditions were applied to subsequent experiments. To search for the potential transcription factors that mediate delta-secretase mRNA expression, we scanned about 3000 nucleotides upstream of the transcription start site of LMGN gene for putative transcription factors binding consensus sequences using the “alibaba2” program, which predicts the transcription factor binding sites by constructing matrices on the fly from TRANSFAC 4.0 sites. This region was considered to contain the major part of LMGN promoter. The computational analysis unveiled dozens of putative transcription factors and hundreds of possible binding sites. Since expression of delta-secretase is elevated in an age-dependent manner^2,3^, we only chose aging-related transcription factors as indicated in the GenAge Database for in-depth investigation. Among these putative transcription factors, only 6 of them were distributed in the brain and implicated in the aging as well. These were SP-1, AP-1, OCT-1, HSF1, C/EBPα, and C/EBPβ, and most of these exhibited numerous putative binding sites on LGMN promoter. To determine which of these transcription factors is involved in delta-secretase mRNA transcription, we utilized siRNA to deplete individual transcription factors in HEK293 cells. Thereafter we monitored delta-secretase expression levels following OGD/reperfusion treatment. Knockdown of HSF1 and C/EBPα strongly reduced delta-secretase protein levels. Remarkably, C/EBPβ depletion completely eliminated delta-secretase expression (Fig. 1b, middle and lower panels), indicating that these transcription factors play a crucial role in the transcription of delta-secretase. To identify the binding sites on the promoter, we constructed pGL3 luciferase reporter plasmids encoding a series of truncated promoter regions from genomic DNA. The borders of these promoter segments were designed to avoid regions with a high frequency of putative binding sites for HSF1, C/EBPα, and C/EBPβ. Luciferase assay showed that base-pairs −2029 to +3 of the promoter exhibited the highest transcription activity (Supplementary Fig. 1a).

To investigate which transcription factor(s) play the major role in mediating delta-secretase transcription, we examined the luciferase activity with −2029 to +3 promoter in the presence of HSF1, C/EBPα, or C/EBPβ individually or in a combination. After OGD/reperfusion treatment, we found that all of these significantly elevated the promoter activity. Once again, C/EBPβ displayed the strongest effect, which was comparable to that of the combination of all three transcription factors (Supplementary Fig. 1b). On the other hand, promoter activities were decreased about five-fold after C/EBPβ was knocked down, followed by C/EBPα with a three-fold reduction. Knocking down of HSF1 conferred the weakest effect on promoter activity (Supplementary Fig. 1c). Mapping experiment demonstrated that C/EBPα and C/EBPβ mainly bound to −2029 to −340 region on the promoter (Supplementary Fig. 1a, d, e). Finally, to ensure that the observed luciferase activity corresponded to delta-secretase mRNA transcription and protein expression, we performed qRT-PCR and immunoblotting analysis. After OGD/reperfusion treatment, delta-secretase mRNA levels were significantly increased following C/EBPα and C/EBPβ overexpression (Supplementary Fig. 2a). On the other hand, when C/EBPα or C/EBPβ was knocked down, the mRNA levels of delta-secretase were repressed accordingly (Supplementary Fig. 2a). Protein levels corresponded to the mRNA levels (Supplementary Fig. 2b).

Next, we wanted to determine whether C/EBPα and C/EBPβ also regulate delta-secretase expression in neurons. Primary neurons were transduced with lentivirus expressing GFP-tagged HSF1, C/EBPα and β, separately or in a combination, at DIV3. After 4 days, the infected neurons were subjected to OGD/reperfusion treatment. qRT-PCR showed that C/EBPα and C/EBPβ, but not HSF1, increased delta-secretase mRNA levels under normoxia, and OGD further escalated this effect. There were no obvious additive effects, when the transcription factors were combined (Fig. 1c). Immunoblotting echoed the observations on the mRNA level. Individually, C/EBPβ overexpression displayed the strongest effect on delta-secretase protein expression under both normoxia and OGD conditions (Fig. 1d, left panels). With a combination of the transcription factors, delta-secretase protein levels were much higher than control regardless of normoxia or OGD stimulation (Fig. 1d, right panels). As expected, depletion of C/EBPα or C/EBPβ, but not HSF1, strongly repressed delta-secretase mRNA under both control and OGD conditions (Fig. 1e). Again, delta-secretase protein expression levels were noticeably inhibited whenever C/EBPβ was depleted, regardless if the neurons were under control or OGD conditions (Fig. 1f, Supplementary Fig. 2c, d), indicating that this transcription factor plays the pivotal role in mediating delta-secretase expression in neurons. Together, these results argue that C/EBPα and β, especially β, are the critical transcription factors regulating delta-secretase transcription in the nervous system.

### C/EBPβ mediates *LGMN* mRNA transcription

**Fig. 2:**
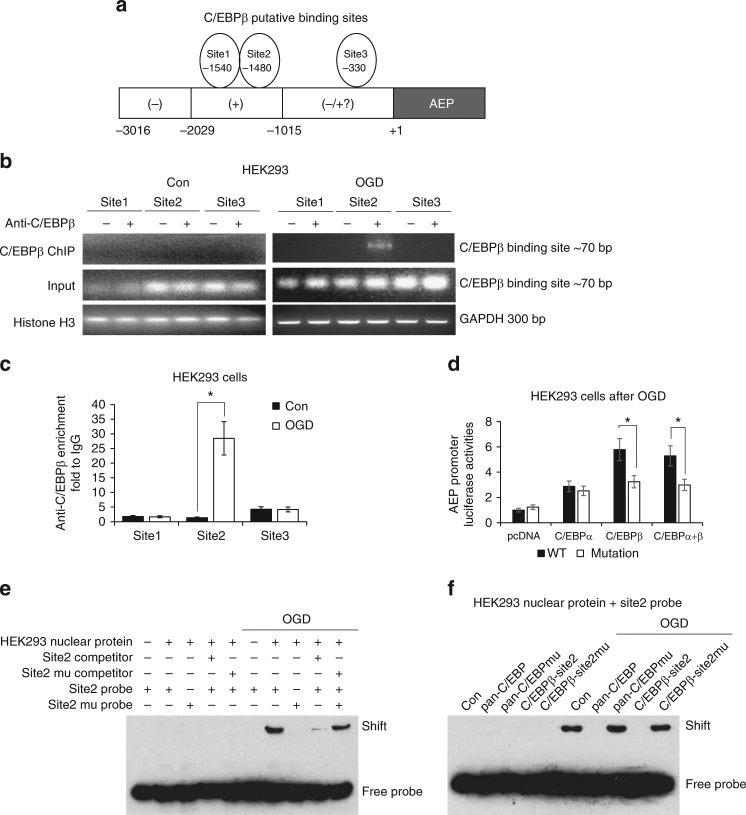
C/EBPβ binds to delta-secretase promoter and regulates its transcription. **a** The model of C/EBPβ putative binding sites on human AEP promoter. **b**, **c** ChIP assay was performed to detect the binding sites of C/EBPβ on the AEP promoter. The DNA-protein crosslinking ChIP samples from HEK293 cells treated with OGD or not, were immunoprecipitated with anti-C/EBPβ antibody or IgG. After reversing crosslinks, PCR (**b**) and real-time PCR (**c**) were performed by using primer pairs at −1540 (site 1), −1480 (site 2), and −330 (site 3) of the human AEP promoter. PCR assay also detected each input sample (**b**). The positive control PCR experiment was done by using anti-Histone H3 antibody and GAPDH primers (**b**). In real-time PCR analysis, the anti-C/EBPβ enrichment was represented by the fold of C/EBPβ antibody enriched target DNA comparing to IgG enriched (**c**, *P < 0.05, Student’s *t*-test). **d** HEK293 cells were transfected with pcDNA, C/EBPα, and/or C/EBPβ, as well as human AEP promoter luciferase reporter plasmid (−2029 to +3) or its mutation (Site 2 was mutant). Forty-eight hours later, OGD/reperfusion was performed and then promoter activities were determined by luciferase assay (*P < 0.05, Student’s *t*-test). **e** Nuclear proteins were isolated from HEK293 cells received OGD/recovering treatment or not. EMSA assay was recruited to detect the C/EBPβ binding ability increasing on Site 2 of AEP promoter after OGD/reperfusion treatment. **f** HEK293 cells were transfected with pan-C/EBP decoy DNA or its mutation, and Site2 decoy DNA or its mutation. Forty-eight hours later, cells were treated with OGD/recovering or not. EMSA assay were then performed to detect C/EBPβ binding abilities on Site 2 of LGMN promoter within each sample of nuclear protein. Data are representative of three independent experiments

Among the 3000 nucleotides upstream of the transcription start site of the LMGN gene, a computational analysis predicted three putative C/EBPβ binding consensus sequences, which were located around −1540 (Site 1), −1480 (Site 2), and −330 (Site 3) (Fig. 2a). To determine which of the three putative binding sites binds to C/EBPβ, we performed a ChIP assay after OGD treatment, and found that only Site 2 strongly bound to C/EBPβ (Fig. 2b, c). To further verify this interaction, we constructed a mutated human delta-secretase promoter luciferase reporter plasmid (containing the −2029 to +3 region), in which the −1480 putative C/EBPβ binding sequence (CCCAGCTCTGGC) was replaced by an EcoRI recognition sequence (GAATTCAGAGGC). As expected, C/EBPβ exhibited much greater activity than that of C/EBPα with the wild-type promoter. However, C/EBPβ overexpression in the presence of the mutant promoter resulted in significantly reduced signal, whereas C/EBPα transcriptional activity did not differ between the wild-type and mutant promoters (Fig. 2d). These data show that this promoter sequence is crucial for C/EBPβ’s transcriptional activity. To further confirm that C/EBPβ binds to the Site 2 motif of the human AEP promoter sequence, we conducted an electrophoretic mobility shift assay (EMSA) assay and found that the mutant −1480 probe failed to bind C/EBPβ in HEK293 nuclear fraction. Moreover, the mutated Site 2 DNA competitor was unable to block the binding of nuclear proteins to the probe, whereas a wild-type Site 2 competitor inhibited the interaction (Fig. 2e). EMSA analysis showed that wild-type decoy DNA oligomers completely blocked C/EBPβ in the nuclear fraction binding to Site 2 probe, whereas mutated pan-C/EBP and C/EBP-Site 2 decoy DNA oligomers failed (Fig. 2f). Hence, the decoy assay verified that C/EBPβ promotes delta-secretase mRNA transcription through binding to the Site 2 region. Collectively, our data strongly support that C/EBPβ acts as a major transcription factor for delta-secretase mRNA transcription through binding to Site 2 motif in the promoter.

### C/EBPβ regulates AEP expression in the CNS in a time-dependent way

Recently, we reported that delta-secretase protein levels increase in an age-dependent manner in the mouse brain and spinal cord^2^. However, it remains unknown whether delta-secretase protein and mRNA expression oscillate in the same temporal pattern. To address these questions, we prepared primary cortical neurons and analyzed delta-secretase levels following different incubation periods in vitro (DIV). As compared to DIV3, primary neuronal cultures at DIV7 demonstrated an evident increase in both delta-secretase protein and mRNA levels, which remained stable thereafter (Supplementary Fig. 3a, b). Overexpression of either C/EBPα or C/EBPβ resulted in significantly enhanced levels of delta-secretase mRNA at DIV7 as compared to DIV3, and the increase in delta-secretase mRNA and protein was even higher with C/EBPβ overexpression (Supplementary Fig. 3c). Deletion of either transcription factor substantially decreased delta-secretase mRNA and protein levels (Supplementary Fig. 3d). Furthermore, subcellular fractionation demonstrated that both C/EBPα and β protein levels escalated in the nuclear fractions over time in neurons. Notably, the elevation in nuclear C/EBPβ over time was more pronounced, which corresponded to the changes seen in delta-secretase expression pattern (Supplementary Fig. 3e). These findings support that C/EBPα and C/EBPβ mediate delta-secretase expression in primary neurons during maturation.

**Fig. 3:**
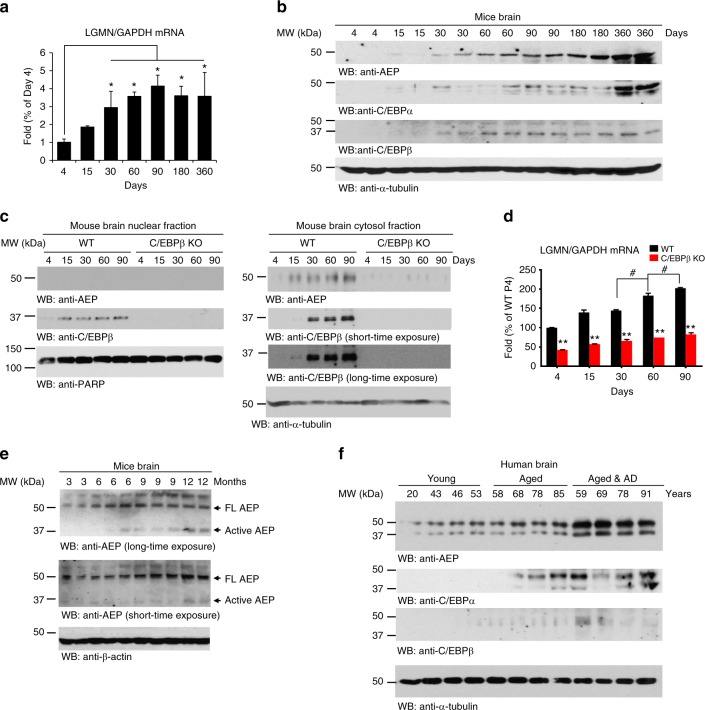
The age-dependent increase of delta-secretase and C/EBPs. **a**, **b** Delta-secretase expression levels correlate with C/EBPβ in mice. Different ages of C57BL/6 mice were analyzed by qRT-PCR and immunoblotting. Data in **a** represent mean ± s.e.m. of six mice per age; *P < 0.05, Student’s *t*-test. **c**, **d** Subcellular fractionation analysis of delta-secretase and C/EBPβ in wild-type and C/EBPβ-null mice. Different ages of wild-type and C/EBPβ-deficient mice were sacrificed and their brains were subjected to subcellular fractionation to obtain the cytosolic and nuclear fractions. Delta-secretase protein (**c**) and mRNA (**d**) levels were analyzed by western blotting and real-time PCR. Delta-secretase predominantly resided in the cytosolic fractions, and depletion of C/EBPβ diminished its expression (**c** top panel). C/EBPβ distributed in both fractions in an age-dependent manner (**c** middle panel). Data in **d** represent mean ± s.e.m. of six mice per age (**P < 0.01 compared with age-matched WT, Student’s *t*-test; ^#^*P* < 0.05 compared with 60-day-old C/EBPβ KO mice, one-way ANOVA). **e**, **f** Delta-secretase increases with age in mouse and human brain. Delta-secretase was cleaved and activated age-dependently in mouse brains (**e**). The expression levels of C/EBPα and C/EBPβ were also detected. The levels of these proteins were much more abundant in the brains of AD patients as compared to age-matched healthy controls (**f**)

To identify the age when delta-secretase mRNA levels start to rise, we quantified delta-secretase mRNA from 4, 15, 30, 60, 90, 180, and 360 days old mouse brains. Detectable delta-secretase mRNA was seen as early as 4 days postnatally, and mRNA levels continued to rise in an age-dependent manner until it plateaued at 90 days (Fig. 3a). Fitting with these observations, delta-secretase protein levels progressively increased with age; as did the protein levels of both C/EBPα and C/EBPβ (Fig. 3b) which is consistent with previous reports demonstrating that both transcription factors are implicated in aging processes^27,28,31,32^. We observed the similar age-dependent upregulation of delta-secretase in mouse kidneys as well (Supplementary Fig. 3f). Moreover, subcellular fractionation showed that nuclear C/EBPβ and cytosolic delta-secretase increased with age in wild-type mice. In contrast, the age-dependent upregulation of delta-secretase mRNA and protein was completely abolished in C/EBPβ KO mouse brains (Fig. 3c, d). Delta-secretase proteolytic cleavage and activation increased with age (Fig. 3e). Furthermore, we detected a significant increase in delta-secretase protein, and its truncated active fragment, in aged human brains. Notably, delta-secretase protein levels were more abundant in brain lysates of AD patients than in age-matched healthy controls. This finding aligned well with the concomitant increase in the expression of both C/EBPα and β (Fig. 3f). Finally, we determined α-Synuclein^33^ and MAO-B mRNA and protein levels, two other reported downstream targets of C/EBPβ, in AD and other neurodegenerative disease such as PD and PSP. There were no significant differences of α-Synuclein and MAO-B levels in AD and age-matched control, though both were elevated in PD (Supplementary Fig. 4), indicating that those targets of C/EBPβ may not be involved in AD.

### C/EBPβ regulates Aβ-induced delta-secretase expression

**Fig. 4:**
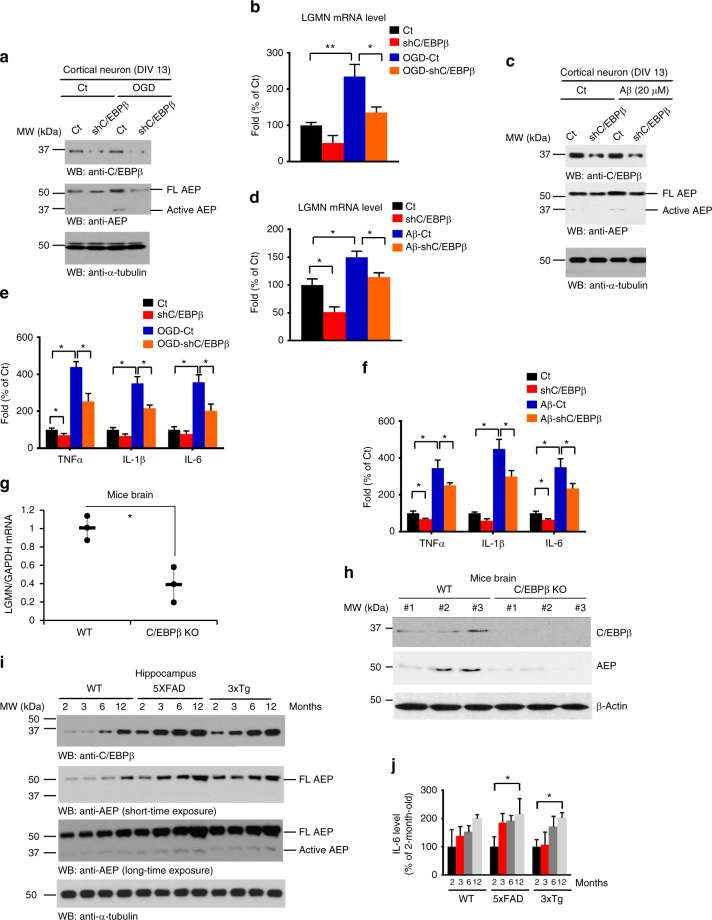
Depletion of C/EBPβ diminishes OGD or Aβ-induced delta-secretase expression. **a**, **b** Knockdown of C/EBPβ reduces delta-secretase expression induced by OGD. Immunoblotting and real-time PCR were conducted from primary cultures infected with virus expressing control or shRNA against C/EBPβ. **c**, **d** Knockdown of C/EBPβ reduces delta-secretase expression induced by Aβ. Western blot data in **a**, **b** are representative of three independent experiments. Data in **c**, **d** represent mean ± s.e.m. of three independent experiments (*P < 0.05, **P < 0.01, one-way ANOVA). **e**, **f** OGD and Aβ stimulate inflammatory cytokine expression, and knockdown of C/EBPβ reduces cytokine levels. Data represent mean ± s.e.m. of three independent experiments (*P < 0.05, one-way ANOVA). **g**, **h** Knockout of C/EBPβ significantly reduces delta-secretase expression in the brain. Wild-type or C/EBPβ KO mice (4–5 months old) were sacrificed and their brains were isolated. Delta-secretase’s mRNA (**g**) and protein (**h**) levels in the brains were analyzed by western blotting and real-time PCR. C/EBPβ knockout was confirmed by immunoblotting. Data in **g** represent mean ± s.d. of three mice per group; *P < 0.05, Student’s *t*-test. **i** Immunoblotting analysis of delta-secretase and C/EBPβ in age-matched wild-type, 5XFAD and 3xTg AD mice. Wild-type and AD mice of different ages were sacrificed and their brain lysates were subjected to western blotting analysis. **j** IL-6 is escalated in AD mouse models in an age-dependent manner. Wild-type and AD mice of different ages were sacrificed and their brain lysates were subjected to IL-6 ELISA analysis. Data represent mean ± s.e.m. of three independent experiments (*P < 0.05, one-way ANOVA)

C/EBPα-null mice die shortly after birth^34^ and C/EBPα is not detectable in normal brains in the absence of injury^35,36^. In contrast, C/EBPβ-deficient mice are viable but display immune defects^37,38^. As demonstrated above, C/EBPβ displayed a much stronger effect on delta-secretase mRNA transcription than C/EBPα, therefore, we chose to focus the remainder of the experimentation on C/EBPβ. Elimination of C/EBPβ significantly diminished OGD-induced delta-secretase protein and mRNA levels (Fig. 4a, b). Depletion of C/EBPβ completely abolished this Aβ-induced increase in delta-secretase (Fig. 4c, d). In alignment with these observations, both OGD and Aβ stimulation increased the expression of inflammatory factors including IL-1, IL-6, and TNF-α. Silencing C/EBPβ substantially repressed this increase in cytokines transcription (Fig. 4e, f). Next, we extended our studies to C/EBPβ knockout mice, and found that delta-secretase transcription and protein expression levels were largely decreased in C/EBPβ KO mice as compared to wild-type littermates (Fig. 4g, h). We found that both C/EBPβ and delta-secretase levels were increased in an age-dependent manner, and C/EBPβ levels tightly correlated with the escalation of delta-secretase temporally. The protein levels of C/EBPβ and delta-secretase in AD mice were clearly higher than in wild-type controls (Fig. 4i), which is consistent with previous reports that describe an upregulation of C/EBPβ and delta-secretase in human AD brains^2,3,22,23^. Inflammatory cytokines were gradually elevated as C/EBPβ levels progressively increased with aging. As expected, the concentrations of the cytokine IL-6, the major downstream effect of C/EBPβ, were significantly higher in 12 months old than in 2 months old AD mouse model brains (Fig. 4j). Together, these findings support that C/EBPβ plays an essential role in mediating Aβ-induced delta-secretase expression and its expression in AD mouse brains in an age-dependent way.

### C/EBPβ mediates AD-like pathogenesis via delta-secretase in 3xTg mice

**Fig. 5:**
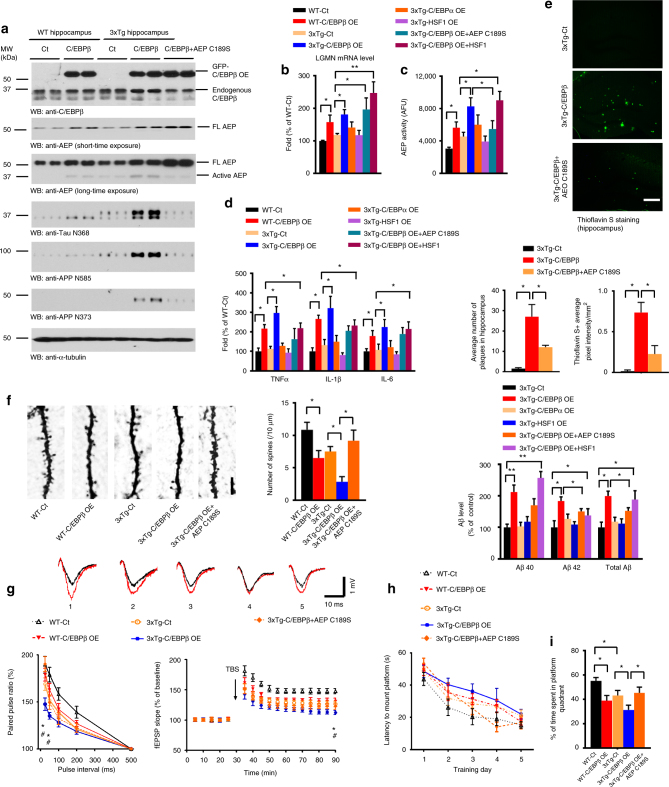
Overexpression of C/EBPβ in young 3xTg mice accelerates the onset of AD-like pathogenesis and worsens cognitive dysfunctions. **a** Overexpression of C/EBPβ escalates delta-secretase and APP and Tau cleavage. Hippocampal tissues were analyzed by immunoblotting. C/EBPβ-induced active delta-secretase was antagonized by the co-expression of C189S mutant (*n* = 3 mice per group). **b**, **c** Overexpression of C/EBPβ but not C/EBPα increases delta-secretase mRNA and enzymatic activities. Data represent mean ± s.e.m. of three mice per group (*P < 0.05, **P < 0.01, one-way ANOVA). **d** Overexpression of C/EBPβ but not C/EBPα increases the expression of inflammatory cytokines. Data represent mean ± s.e.m. of three mice per group (*P < 0.05, one-way ANOVA). **e** C/EBPβ overexpression enhances the early formation of amyloid plaques in 3xTg mice. Thioflavin S staining for the senile plaques (top, scale bar, 50 μm). Analysis data (middle) represent mean ± s.e.m. of 12–18 sections from three mice per group and Aβ ELISA (lower) represent mean ± s.e.m. of three mice per group (*P < 0.05, **P < 0.01, one-way ANOVA). **f** Overexpression of C/EBPβ decreases dendritic spine density. Golgi staining was conducted on brain sections from apical dendritic layer of the CA1 region. Scale bar, 5 μm. Data (right) represent mean ± s.e.m. of 9–12 sections from three mice in each group. (*P < 0.05, one-way ANOVA). **g** Electrophysiology analysis. C/EBPβ overexpression worsened the LTP defects in 3xTg mice. The ratio of paired pulses (mean ± s.e.m.; *n* = 6 in each group; *P < 0.05 compared with 3xTg-Ct, ^#^*P* < 0.05 compared with 3xTg-C/EBPβ+AEP C189S, one-way ANOVA) (left). LTP of fEPSPs (mean ± s.e.m.; *n* = 6 in each group; *P < 0.05 compared with 3xTg-Ct, ^#^*P* < 0.05 compared with 3xTg-C/EBPβ+AEP C189S, one-way ANOVA) (right). Shown traces are representative fEPSPs of 10 samples recorded before (black) and after (red) TBS (theta-burst stimulation). 1, WT-Ct; 2, WT-C/EBPβ OE; 3, 3xTg-Ct; 4, 3xTg-C/EBPβ OE; 5, 3xTg-C/EBPβ+C189S AEP. **h**, **i** Morris water maze analysis. C/EBPβ overexpression exacerbated the learning and memory dysfunctions (mean ± s.e.m.; *n* = 7–8 mice per group; *P < 0.05, one-way ANOVA)

Delta-secretase cleaves both APP and Tau in human AD brains^2,3^ and C/EBPβ is increased in human AD brains^39^. We thus hypothesized that increased C/EBPβ expression would augment delta-secretase transcription and protein levels, resulting in earlier onset of pathological defects in AD mouse models. The triple-transgenic model 3xTg AD mice (APPswe, PS1_M146V_, Tau P301L) progressively develop plaques and tangles. Synaptic dysfunction, including LTP deficits, manifests in an age-related manner, but before plaque and tangle pathology (~12 months)^40^. To test our hypothesis, we injected lentivirus (LV) expressing C/EBPβ into the hippocampus of 3 months old 3xTg mice and age-matched wild-type mice (Supplementary Table 1). In addition, we also injected AAV expressing a dominant-negative form of delta-secretase (C189S) to test whether this form might inhibit C/EBPβ’s effects in 3xTg mice. Overexpression of C/EBPβ resulted in increased delta-secretase expression in wild-type mice that was associated with increased levels of cleaved active delta-secretase as compared with control. Noticeably, untreated 3xTg mice expressed higher levels of delta-secretase as compared to wild-type controls; this increase was also accompanied with higher levels of the active form of delta-secretase. The levels of both full-length and cleaved delta-secretase were further elevated due to C/EBPβ overexpression in 3xTg mice (Fig. 5a, top three panels). APP N373, N585, and Tau N368 proteolytic fragments by delta-secretase were increased as a result of C/EBPβ overexpression, and their expression patterns mirrored the expression manners of active delta-secretase (Fig. 5a, 4th—bottom panel; Supplementary Fig. 6a). Importantly, expression of C189S mutant strongly reduced this proteolytic activity, indicating that C/EBPβ-induced fragmentation of both APP and Tau is mediated by delta-secretase (Fig. 5a, lanes 9 and 10). Delta-secretase mRNA levels and enzymatic activity were also enhanced by C/EBPβ but not C/EBPα (Fig. 5b, c). C/EBPβ overexpression in 3xTg mice resulted in an increase in the production of inflammatory cytokines than that seen in wild-type mice, whereas these factors remained similar between wild-type and 3xTg mice in the absence of C/EBPβ overexpression (Fig. 5d). Again, expression of the C189S mutant significantly inhibited C/EBPβ-elicited delta-secretase enzymatic activity and the induction of cytokines (Fig. 5c, d). Thioflavin S staining demonstrated that amyloid plaques in the hippocampus were more numerous and bigger in C/EBPβ overexpressing 3xTg mice to control-treated animals (Fig. 5e). Aβ ELISA analysis demonstrated that C/EBPβ but not C/EBPα greatly increased Aβ in 3xTg mice (Fig. 5e, bottom panel). This finding was confirmed by immunohistochemistry using an anti-Aβ antibody (Supplementary Fig. 5a). Again, co-expression of the C189S mutant substantially blunted C/EBPβ-mediated increase in plaque formation in the hippocampus. In alignment with the increase in cytokine production with C/EBPβ overexpression, a marked induction of microglial activation in 3xTg mice was seen by Iba1 immunoreactivity. Expression of the dominant-negative delta-secretase C189S reduced the degree of microglial activation (Supplementary Fig. 5b). Electron microscopy (EM) revealed that C/EBPβ overexpression significantly reduced the number of synapses in 3xTg as compared to wild-type mice (Supplementary Fig. 5c). A quantitative analysis of Golgi staining showed a similar pattern where hippocampal spine density was decreased in C/EBPβ overexpressing animals (Fig. 5f). Again, these C/EBPβ-induced ultrastructural defects were both reversed by co-expression of C189S.

**Fig. 6:**
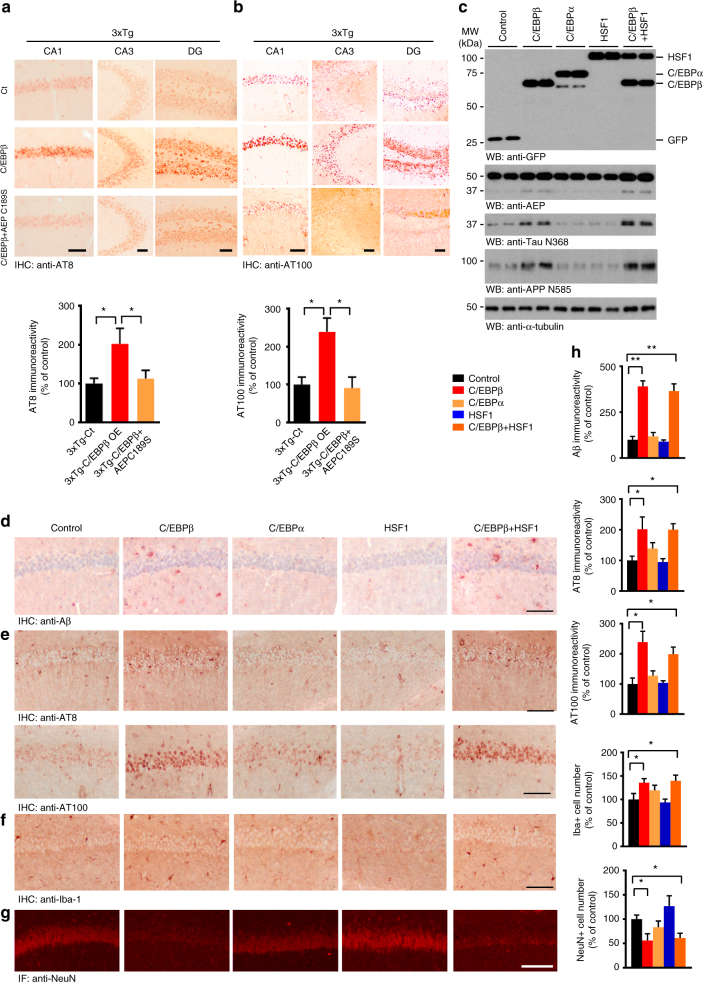
Specificity of C/EBPβ overexpression in accelerating AD-like pathogenesis. **a**, **b** AT8 and AT100 IHC staining of Tau pathology in different hippocampal regions. Overexpression of C/EBPβ significantly escalated Tau hyperphosphorylation in 3xTg mice. Data represent mean ± s.e.m. of 12–18 sections from three mice in each group (*P < 0.05, one-way ANOVA). Scale bar, 100 μm. **c** Overexpression of C/EBPα or HSF1 does not escalate delta-secretase and APP and Tau cleavage by delta-secretase. Hippocampal tissues from both control and virus injected 3xTg mice were analyzed by immunoblotting. C/EBPβ-induced active delta-secretase was not antagonized by the co-expression of HSF1, an unrelated transcription factor (*n* = 4 mice per group). **d**–**g** Immunostaining of Aβ, AT8, AT100, Iba-1, and NeuN in hippocampal CA1. **h** Quantifications of Aβ, AT8, AT100, Iba-1, and NeuN immuno-reactivities represent mean ± s.e.m. of 12–18 sections from three mice in each group (*P < 0.05, one-way ANOVA). Scale bar, 150 μm

Long-term potentiation (LTP), a form of plasticity, is thought to underlie learning and memory^41^. LTP is significantly reduced in 3xTg mice compared to Non-Tg mice at 6 months of age^40^. Electrophysiologic measurements within the CA1 region of the hippocampus demonstrated that C/EBPβ expression reduced the synaptic plasticity in both 3xTg and wild-type mice, but LTP was much lower in 3xTg mice than wild-type (Fig. 5g; Supplementary Fig. 5d). Morris water maze (MWM) behavioral testing demonstrated that 3xTg/C/EBPβ mice displayed the biggest impairment in learning and memory, followed by wild-type/C/EBPβ. In alignment with previous reports^42^, 3xTg/control exhibited cognitive defects as compared with wild-type/controls. However, it is worth noting that C/EBPβ overexpression impaired cognition in both wild-type and 3xTg mice as compared to mice of either genotype treated with the control virus. Strikingly, expression of C189S mutant rescued the C/EBPβ-mediated cognitive impairments in 3xTg mice (Fig. 5h, i; Supplementary Fig. 5e), although the swim speed remained the same between these groups (Supplementary Fig. 5f). A fear conditioning test showed that both contextual and cued fear conditioning were significantly reduced with LV-C/EBPβ overexpression in 3xTg mice, an effect which was completely rescued by C189S co-expression (Supplementary Fig. 5g, h). AT8 and AT100 staining showed that LV-C/EBPβ facilitated Tau phosphorylation and Tau pathology to a greater extent than that seen with control virus-treated 3xTg mice. Tau phosphorylation and the associated pathology was absent in AEP C189S-treated animals (Fig. 6a, b). It is worth noting that C/EBPβ overexpression elicited prominent NeuN reduction in 3xTg mice, indicating extensive neuronal loss, which was reversed by inactive AEP C189S (Supplementary Fig. 6b). To investigate whether these events were specifically mediated by C/EPBβ, we overexpressed other transcription factors, C/EBPα or HSF1, via viral injection. Overexpression of C/EBPα or HSF1 in young 3xTg mice did not significantly accelerate the onset of AD-like pathogenesis or worsen cognitive dysfunctions (Fig. 6c–h; Supplementary Fig. 7). Furthermore, there was no significant change in co-expression studies of C/EBPβ with another LV expressing HSF1 in 3xTg mice compared with overexpressing C/EBPβ only (Fig. 6c–h; Supplementary Fig. 7), eliminating the possibility that co-expression may reduce the effect of C/EBPβ. Therefore, our data strongly support that C/EBPβ escalates the expression of delta-secretase, and triggers APP and Tau cleavage by delta-secretase, ultimately leading to neuronal cell loss and earlier onset of AD-like pathogenesis in 3xTg and exacerbation of cognitive impairments. Inhibition of delta-secretase by co-expression of the dominant-negative C189S mutant substantially ameliorated these C/EBPβ-induced effects.

### Reduction of C/EBPβ decreases delta-secretase and AD-like pathologies

**Fig. 7:**
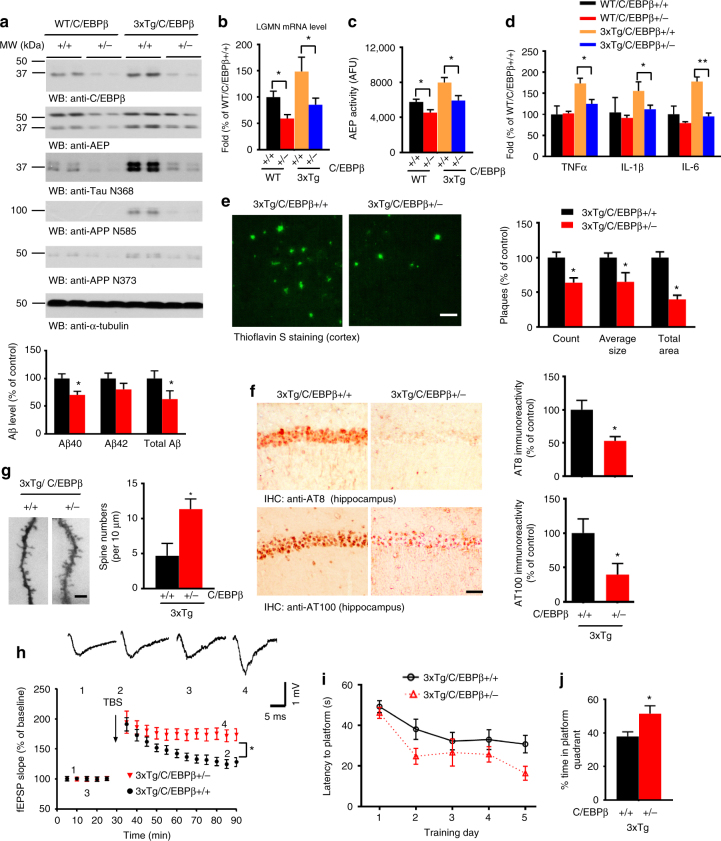
Reduction of C/EBPβ gene dose reverses AD-like pathologies and alleviates cognitive impairments in old 3xTg mice. **a** Reduction of C/EBPβ decreases delta-secretase and APP and Tau cleavage by delta-secretase. The hippocampal tissues were analyzed by immunoblotting (*n* = 2 mice per group). **b**, **c** Reduction of C/EBPβ reduces delta-secretase mRNA and enzymatic activities. Data represent mean ± s.e.m. of three mice per group (*P < 0.05, one-way ANOVA). **d** Reduction of C/EBPβ decreases expression levels of inflammatory cytokines. Data represent mean ± s.e.m. of three mice per group (*P < 0.05, **P < 0.01, one-way ANOVA). **e** Amyloid plaques are decreased in 3xTg/C/EBPβ +/− mice. Thioflavin S staining showing the Aβ plaques in the frontal cortex (left panels). Scale bar, 50 μm. Plaque analysis data represent mean ± s.e.m. of 12–19 sections from three mice in each group (right, *P < 0.05, Student’s *t*-test). Aβ ELISA represent mean ± s.e.m. of three mice per group (lower, *P < 0.05, Student’s *t*-test). **f** Tau pathologies are reduced in 3xTg/C/EBPβ +/− mice. The brain sections of 3xTg and 3xTg/C/EBPβ +/− mice (10 months old) were immunostained with AT8 (upper left panels) and AT100 antibody (lower left panels) (mean ± s.e.m.; 12–18 sections from three mice, *P *<* 0.05, Student’s t-test). Scale bar, 50 μm. **g** Spine density in the hippocampus determined by Golgi staining (mean ± s.e.m.; *n* = 4; *P < 0.05, Student’s *t*-test). Scale bar, 5 μm. **h** Electrophysiology analysis. C/EBPβ reduction in the hippocampus rescued the LTP defects in aged 3xTg mice (mean ± s.e.m.; n = 6 in each group; *P < 0.05, Student’s t-test). Shown traces are representative fEPSPs of four samples recorded at the time points 1 and 2 (3xTg/C/EBPβ +/+) and 3 and 4 (3xTg/C/EBPβ +/−). **i**, **j** Morris water maze analysis of cognitive functions. C/EBPβ reduction in the hippocampus rescues the learning and memory impairments in 3xTg mice (mean ± s.e.m.; *n* = 8 mice per group; *P < 0.05, Student’s *t*-test)

To assess whether reduction of C/EBPβ in this mouse model will suppress delta-secretase expression, delaying the onset of the pathological and behavioral defects, we crossed C57BL/6^C/EBPβ^ +/− with 3xTg mice and analyzed the associated pathologies and cognitive behaviors at 10–11 months of age. C/EBPβ was elevated in 3xTg mice as compared to age-matched wild-type mice, but C/EBPβ levels were obviously reduced in 3xTg/C/EBPβ +/− mice (Fig. 7a, top panel). Accordingly, delta-secretase mRNA and enzymatic activity were significantly reduced in 3xTg/C/EBPβ +/− as compared to 3xTg mice (Fig. 7b, c). We also observed the corresponding levels of active (cleaved) delta-secretase protein levels in these mice (Fig. 7a, 2nd panel). As expected, its proteolytic cleavage products, including the Tau N368, APP N585, and APP N373 fragments, were all reduced in 3xTg/C/EBPβ +/− mice (Fig. 7a, 3-bottom panels; Supplementary Fig. 8h). Moreover, the production of inflammatory cytokines was significantly attenuated in 3xTg/C/EBPβ +/− mice (Fig. 7d). Thioflavin S staining and Aβ ELISA analysis demonstrated a much lower deposition of amyloid plaques deposited in the cortex and a reduction in the levels of Aβ peptides in 3xTg/C/EBPβ +/− mice (Fig. 7e). IHC staining with anti-Aβ validated these observations (Supplementary Fig. 8a). Remarkably, p-Tau measured with both AT8 and AT100 antibodies revealed that neurofibrillary tangles were noticeably reduced in 3xTg/C/EBPβ +/− mice (Fig. 7f). We also observed a marked reduction in microglial activation in 3xTg/C/EBPβ +/− mice versus 3xTg mice (Supplementary Fig. 8b). Quantifications of dendritic spines and synapses were significantly elevated in 3xTg/C/EBPβ +/− mice (Fig. 7g, Supplementary Fig. 8c). LTP was significantly elevated in 3xTg/C/EBPβ +/− mice as compared to 3xTg mice (Fig. 7h; Supplementary Fig. 8d). MWM behavioral test demonstrated that the latency to reach the submerged platform was lessened in C/EBPβ heterozygous mice. Accordingly, they spent markedly longer time in the quadrant where the platform was located (Fig. 7i, j; Supplementary Fig. 8e). Both genotypes exhibited comparable swim speed, indicating that C/EBPβ gene reduction does not affect motor functions (Supplementary Fig. 8f). The contextual fear assay demonstrated that 3xTg/C/EBPβ +/− mice display improved memory as compared to 3xTg mice (Supplementary Fig. 8g). Complete knockout of C/EBPβ from 3xTg mice also reduces delta-secretase expression and rescues impairment of synaptic plasticity (Supplementary Fig. 9). These data support that a reduction in C/EBPβ attenuates delta-secretase expression and the resulting AD-like pathology, and largely reverses spatial and associative learning deficits in old 3xTg mice.

### C/EBPβ mediates AD-like pathologies in 5XFAD mice

**Fig. 8:**
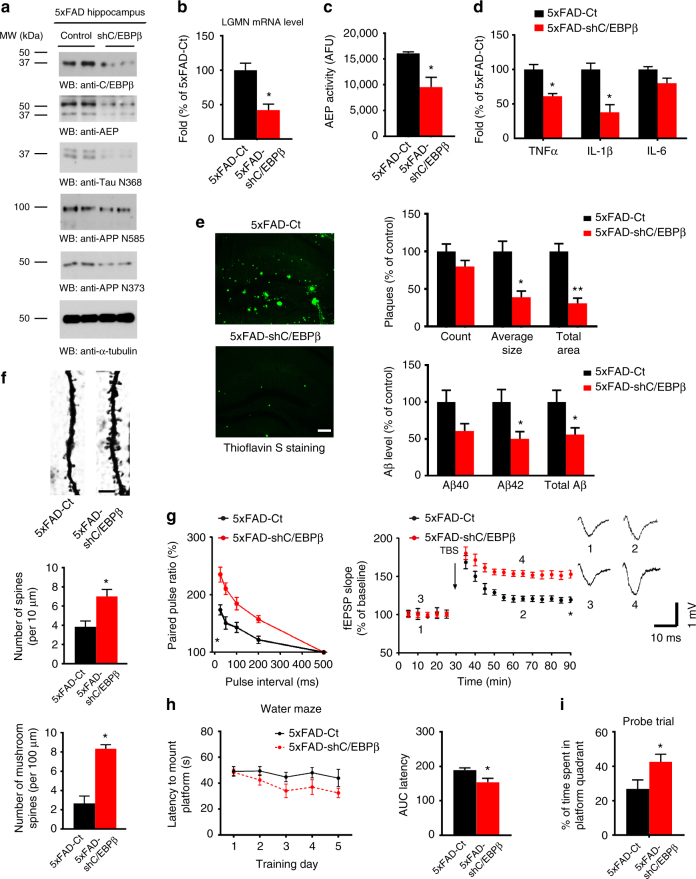
Knockdown of C/EBPβ in 5XFAD mice reduces AD-like pathogenesis and rescues cognitive function. **a** Knockdown of C/EBPβ reduces delta-secretase and APP and Tau fragments cleaved by delta-secretase. Hippocampal tissues from both control and C/EBPβ−shRNA injected 5XFAD mice were analyzed by immunoblotting with various antibodies (*n* = 3 mice per group). **b**, **c** Knockdown of C/EBPβ reduces delta-secretase mRNA and enzymatic activities. Data represent mean ± s.e.m. of three mice per group (*P < 0.05, Student’s *t*-test). **d** Knockdown of C/EBPβ decreases expression levels of inflammatory cytokines. Data represent mean ± s.e.m. of three mice per group (*P < 0.05, Student’s *t*-test). **e** C/EBPβ knockdown decreases amyloid plaques and Aβ in 5XFAD mice. Quantification of number and surface area of amyloid plaques as well as measurements of Aβ levels by ELISA (right panels). Plaque analysis data represent mean ± s.e.m. of 11–17 sections from three mice in each group (*P < 0.05, **P < 0.01, Student’s *t*-test). Aβ ELISA represents mean ± s.e.m. of three mice per group (*P < 0.05, Student’s *t*-test). Scale bar, 50 μm. **f** Knockdown of C/EBPβ increases the spine density in the hippocampal neurons. Scale bar, 5 μm. Golgi staining was conducted on brain sections from control and C/EBPβ-shRNA injected hippocampal regions of 5XFAD mice (mean ± s.e.m.; *n* = 5; *P < 0.05, Student’s *t*-test). **g** Electrophysiology analysis. C/EBPβ silencing in the hippocampus rescued the LTP defects in 5XFAD mice (mean ± s.e.m.; *n* = 6 in each group; *P < 0.05, Student’s *t*-test). Shown traces are representative fEPSPs of four samples recorded at the time points 1 and 2 (5XFAD-Ct) and 3 and 4 (5XFAD-shC/EBPβ) mice. **h**, **i** Morris water maze analysis of cognitive functions. C/EBPβ deletion in the hippocampus rescues the learning and memory impairments in 5XFAD mice (mean ± s.e.m.; *n* = 8 mice per group; *P < 0.05, Student’s *t*-test)

Knockout of delta-secretase in 5XFAD mice reduces senile plaques, ameliorates synapse loss, and rescues learning and memory functions^3^. We thus hypothesized that if C/EBPβ is the key transcription factor for delta-secretase, silencing its expression should induce similar results. Accordingly, we injected LV expressing a C/EBPβ shRNA into the hippocampus of 2.5 months old 5XFAD mice that present early and severe amyloid pathology. Three months later we conducted a panel of assays to systemically analyze the effects of C/EBPβ knockdown. As expected, C/EBPβ shRNA expression decreased the protein and mRNA levels of delta-secretase by approximately 50% (Fig. 8a, b). As a result, the overall enzymatic activity of delta-secretase was attenuated as well (Fig. 8c). As a consequence, delta-secretase-mediated production of APP (N373 and N585) and Tau (N368) fragments were reduced in the hippocampus, along with a reduction in the levels of inflammatory cytokines (Fig. 8a, d). Delta-secretase plays a critical role in mediating APP metabolism and Aβ production, and knockout of delta-secretase results in an approximately 30% reduction of Aβ in 5XFAD mice^3^. To examine the effect of reduced delta-secretase activity on Aβ deposition, we conducted Thioflavin S staining and an Aβ ELISA analysis. We found that amyloid plaque deposition and Aβ levels were significantly reduced in the hippocampus with the reduction of C/EBPβ (Fig. 8e). IHC analysis with anti-Aβ confirmed the ELISA data (Supplementary Fig. 10a). C/EBPβ knockdown was also associated with a reduction in inflammatory factors and activated microglia (Supplementary Fig. 10b). Depletion of C/EBPβ increased the number of synapses in 5XFAD mice as compared with control (Supplementary Fig. 10c). Golgi staining demonstrated increased spine density in shC/EBPβ-virus-treated mice (Fig. 8f). 5XFAD mice display significantly impaired LTP at Schaffer collateral-CA1 pathways^43^. Accordingly, an electrophysiological analysis showed that synaptic plasticity was elevated, when C/EBPβ was silenced in 5XFAD mice (Fig. 8g). MWM behavioral test demonstrated that the latency to reach the submerged platform was substantially decreased with C/EBPβ-shRNA treatment as compared to control-treated mice. Consequently, the time spent in the correct quadrant was increased (Fig. 8h, i). Both strains of mice exhibited comparable swim speeds (Supplementary Fig. 10d). Next, we performed cued fear conditioning assay and found that C/EBPβ-depleted mice exhibited enhanced memory in cued fear freezing test as compared to control mice (Supplementary Fig. 10e). To demonstrate the specificity of C/EPBβ, we evaluated the effect of knockdown of C/EBPα or HSF1 in 5XFAD hippocampus via virus injection. Knockdown of C/EBPα or HSF1 in 5XFAD mice did not significantly reduce AD-like pathogenesis and cognitive functions (Supplementary Fig. 11). Hence, knockdown of C/EBPβ selectively reduces delta-secretase expression and subsequent AD-like pathologies, reversing the spatial and associative learning defects in 5XFAD mice.

## Discussion

In the current study, we found that C/EBPβ acts as a crucial transcription factor for delta-secretase, and regulates its mRNA transcription and protein expression in an age-dependent manner. Remarkably, knockdown of C/EBPβ in primary neurons greatly diminishes delta-secretase levels and the induction of inflammatory cytokines by OGD or Aβ. 3xTg-AD mice progressively develop Aβ and Tau pathology with a temporal- and regional-specific profile that closely mimics the development of pathology in the human AD brain. Aβ deposits initiate in the cortex and spreads to the hippocampus with aging, whereas Tau pathology is first apparent in the hippocampus and then gradually progresses to the cortex^40^. At 6 months of age, extracellular Aβ deposits are evident only in the neocortex, and not in the CA1 region of the hippocampus of 3xTg. However, no Tau alterations are apparent at this age. It is not until the 3xTg mice reach about 12 months of age that extensive human Tau immune-reactivity is evident in CA1 neurons^40^. Hence, this temporo-spatial discrepancy in amyloid deposition and Tau pathology provide us with a great opportunity to explore the pathological roles of C/EBPβ in the hippocampus in 3xTg mice. Remarkably, overexpression of C/EBPβ in the hippocampus of young 3xTg mice enhances delta-secretase expression and accelerates earlier onset of AD-like pathogenesis. Strikingly, co-expression of a dominant-negative form of delta-secretase (C189S) rescues these defects triggered by C/EBPβ overexpression, supporting our hypothesis that C/EBPβ-elicited pathological impairments are mediated via upregulating delta-secretase.

Aging is the major risk factor for AD. Both C/EBPα and C/EBPβ are sufficient and necessary for delta-secretase mRNA transcription in HEK293 cells and primary cultured neurons under OGD, a model to mimic the chronic cerebral hypo-perfusion that occurs during the aging process. Aβ also increases both delta-secretase and inflammatory cytokines in a C/EBPβ-dependent manner in primary neurons to the same extent as OGD does. Conceivably, Aβ deposition during the aging process may activate C/EBPs, promoting the transcription of both delta-secretase and inflammatory cytokines in AD.

The transcription factors C/EBP α, β, and δ have been shown to be expressed in the brain and implicate in regulation of inflammatory genes in concert with nuclear factor κB (NF-κB). In general, C/EBPα is downregulated, whereas both C/EBPβ and δ are up-regulated in response to inflammatory stimuli. Interestingly, both expression and function of C/EBPδ are dysregulated in AD. C/EBPδ seems to be differently regulated in response to different conformations of Aβ. However, C/EBPβ is upregulated by Aβ in AD^24^. Moreover, IL-17 signal transduction regulates a variety of pro-inflammatory and antimicrobial genes by activating NF-κB and MAPK pathways as well as the C/EBP family of transcription factors, such as C/EBPβ and C/EBPδ^44^. It has been reported that MAPK and GSK3β mediates C/EBPβ activity via sequential phosphorylation of on T188 and T179, respectively. Noticeably, GSK3β phosphorylation of C/EBPβ inhibits its transcriptive activity^45–47^.

In this paper, it clearly demonstrates that ischemia/reperfusion selectively stimulates C/EBPβ isoform but not other isoforms to translocate into the nucleus, promoting IL-6 transcription using EMSA. Conceivably, OGD may selectively trigger C/EBPβ nuclear translocation and bind to *LGMN* promoter, escalating delta-secretase mRNA transcription and protein expression. Focal cerebral ischemia induced by MCAO significantly increases C/EBPβ gene expression in mouse brain between 6 and 72 h of reperfusion^26^. C/EBPβ-null mice show significantly smaller infarcts, reduced neurological deficits, decreased apoptosis, decreased extravagated neutrophils, and fewer activated microglia/macrophages. Moreover, C/EBPβ KO mice exhibit a reduced induction of genes that promote inflammation and neuronal damage following focal ischemia^26^. We have previously reported that focal ischemia activates AEP, which subsequently cleaves SET. Knockout of AEP reduces stroke-mediated neuronal cell death^5^. Imaginably, repression of delta-secretase expression in C/EBPβ KO mice may attribute to the diminished neuronal damage.

C/EBPβ is implicated in neuro-inflammation^16,18–20^. There is a positive feed-back loop between inflammatory components and C/EBPβ in the brain^23^. Furthermore, C/EBPβ is upregulated in the AD brain^39^. Thus, this transcription factor may positively regulate the appearance of major histopathological features of AD such as the formation of senile plaques, NFT, neuronal loss and neuro-inflammation through impinging on the transcription machinery of delta-secretase. Accordingly, knockdown of C/EBPβ in the AD mouse models may interrogate its pathological role in these processes. Indeed, we found that depletion of C/EBPβ in young 5XFAD and old 3xTg mice greatly eliminates delta-secretase expression, leading to inhibition of Aβ production, Tau hyperphosphorylation, and neuro-inflammation. In contrast, overexpression of C/EBPβ in young 3xTg mice resulted in an early and significant enhancement of the major pathological features including extracellular amyloid deposit and production of inflammatory cytokines in the hippocampus. Noticeably, intraneuronal neurofibrillary tangles, which normally does not appear until 12 month old of age or later in 3xTg mice^40^, were formed at about 6 months of age as a result of C/EBPβ overexpression. As a result, synaptic loss-associated LTP defects and cognitive impairments were strongly exacerbated. Furthermore, extensive neuronal loss occurred in young 3xTg mice. Conversely, reduction of C/EBPβ gene (C/EBP+/−) reversed the pathological features and cognitive dysfunctions observed in 3xTg.

Though C/EBPβ-null mice from the mixed strain background (C57BL/6; 129SV) are viable^34^, they are affected by a lymphoproliferative disorder, impaired lipid metabolism in brown fat tissue^38,48^, defective regenerative capacity of hepatocytes, impaired liver function^49,50^. In order to avoid these potential complications, therefore, we chose to compare the pathological differences between 3xTg/C/EBPβ +/−, instead of 3xTg/C/EBPβ −/−, with 3xTg-AD mice. However, C/EBPβ +/− mice do not possess any of these defects and they behave similar to wild-type littermate. Hence, we omitted these two strains in our AD-related pathological and cognitive behavioral study. Our findings establish a novel function to C/EBPβ in AD disease etiology. In addition to mediating neuro-inflammation, C/EBPβ also controls the expression of delta-secretase, an essential protease for neuronal cell death and cleaving both APP and Tau. These striking observations provide convincing evidence revealing the dominant role of the age-dependent transcription factor C/EBPβ in AD onset and progression.

## Methods

### Human samples

Post-mortem brain samples were dissected from frozen brains of AD, PD, and PSP cases and non-demented controls from the Emory Alzheimer’s Disease Research Center. The study was approved by the Biospecimen Committee. AD, PD, and PSP were diagnosed according to the criteria of the Consortium to Establish a Registry for AD, PD, and PSP and the National Institute on Aging. Diagnoses were confirmed by the presence of amyloid plaques and neurofibrillary tangles in formalin-fixed tissue. Informed consent was obtained from the subjects.

### Mice and cells

Wild-type C57BL/6J mice, 3xTg and 5XFAD mice were ordered from the Jackson Laboratory (000664, 34830, 34840). C/EBPβ knockout mice have been described^37^. Since the homozygous mutation is lethal on pure strain backgrounds, *Cebpb* mice were maintained as heterozygotes on two separate strain backgrounds (C57BL/6 and 129Sv). These two strains were crossed to generate viable F1 hybrid WT and *Cebpb−/−* littermates, which were used for aging studies. Both female and male mice were used. Animal care and handling were performed according to NIH animal care guidelines and the Declaration of Helsinki and Emory Medical School guidelines. The protocol was reviewed and approved by the Emory Institutional Animal Care and Use Committee. Primary rat cortical neurons were cultured as previously described. All rats were purchased from the Jackson Laboratory. The protocol was reviewed and approved by the Emory Institutional Animal Care and Use Committee. HEK293 cells were cultured in high-glucose DMEM added with 10% fetal bovine serum (FBS) and penicillin (100 units/ml)—streptomycin (100 μg/ml) (all from Hyclone). Cells were incubated at 37 °C in a humidified atmosphere of 5% CO_2_. δ-secretase antibody (clone 6E3) was a present from Dr. Colin Watts, University of Cambridge. Beta-Actin antibody was purchased from Sigma. Antibody to C/EBPβ (H-7) was from Santa Cruz Biotechnology (Santa Cruz, CA, USA). HSF1, C/EBPα, p-C/EBPβ, PARP antibodies were bought from Cell Signaling Technology. GFP and alpha-tubulin antibodies were purchased from Santa Cruz. AT8 (Thermo Fisher, 1: 1000), AT100 (Thermo Fisher, 1:500), 4G8 (Covance, 1:500), NeuN (Cell Signaling, 1:1000) were used for immunostaining antibodies.

### Transfection and infection of the cells

The overexpressing plasmids were purchased from Addgene. The siRNAs were bought from Santa Cruz. The overexpression lentivirus (pFCGW-GFP) was from the viral vector core (VVC) of Emory University. The shRNA expressing lentivirus for HSF1, C/EBPα, and C/EBPβ were ordered from OriGene Technologies, Inc. (Rockville, MD). HEK293 transfection was performed using Lipofectamine 2000 (Invitrogen). The lentiviruses used in neuron infection and brain injection were packaged in VVC of Emory University. AAV expressing AEP C189S was packaged by the Manfredsson laboratory at Michigan State University

### OGD/reperfusion treatment

Cultured HEK293 cells or primary neurons were rinsed with PBS for three times and re-incubated in DMEM without FBS or glucose. Then cells were introduced into an anaerobic chamber containing a mixture of balanced N_2_, 1% O_2_, and 5% CO_2_ at 37 °C for 10 h (HEK293 cells) or 2 h (primary neurons). Cells in the non-OGD groups were re-incubated in normal DMEM with FBS (HEK293 cells) or without FBS (neurons) at 37 °C in a humidified atmosphere of 5% CO_2_. At the end of OGD, all medium was replaced and cells were re-cultured in normal conditions for 12 h (HEK293 cells) or 24 h (primary neurons) of recovery.

### Western blotting

The cells were lysed in RIPA buffer (20 mM pH 7.5 Tris-HCl, 150 mM NaCl, 1 mM Na_2_EDTA, 1 mM EGTA, 1% Triton, 2.5 mM sodium pyrophosphate, 1 mM beta-glycerophosphate, 1 mM Na_3_VO_4_, 1 µg/ml leupeptin, 1 mM phenylmethylsulfonyl fluoride) on ice for 30 min. Cell lysates were then centrifuged at 13,000×*g* for 30 min at 4 °C. Supernatant was collected, and protein concentration was determined using a Coomassie Brilliant Blue protein assay kit (Bio-Rad). Western blot was carried out and AEP antibody 6E3.

### Real-time PCR

Levels of mRNA were analyzed by real-time, quantitative PCR. RNA was isolated by Trizol (Life Technologies). Reverse transcription was performed with SuperScriptIII reverse transcriptase (Life Technologies). Gene-specific primers and probes were designed and bought from Taqman (Life Technologies). All real-time PCR reactions were performed using the ABI 7500-Fast Real-Time PCR System and Taqman Universal Master Mix Kit (Life Technologies). The relative quantification of gene expression was calculated as 2^−Δ^^Δ^^Ct^ method. For each data point, at lease duplicated wells were used. And each experiment was repeated at least for three times.

### Luciferase assay

HEK293 cells were seeded in 24-well plates and cultured overnight. Cells were then transfected with AEP luciferase reporter together with pRL-TK Renilla luciferase plasmid (Promega), as well as different kinds of overexpressing plasmids or siRNA. Forty-eight hours post transfection, OGD/reperfusion was performed. Then, the cells were harvested in passive lysis buffer and analyzed using a Dual-Luciferase Reporter Assay System according to the manufacturer’s protocol (Promega) on a microplate reader. Relative light units of AEP luciferase were normalized to Renilla luciferase light units to control for transfection efficiency. The experiments were performed in triplicate.

### EMSA (Electrophoretic mobility shift assay; DNA:Protein)

Nuclear proteins of HEK293 were extracted by using NE-PER Nuclear and Cytoplasmic Extraction Reagents (Life Technologies). Protein concentrations were determined using a Coomassie Brilliant Blue protein assay kit (Bio-Rad). Double-stranded oligonucleotide probe for Site2 of AEP promoter or its mutation were labeled with biotin. Unlabeled probes were used as competitors or mutant competitors. EMSA assay was performed as described in LightShift Chemiluminescent EMSA Kit (Life Technologies).

### Decoy assay

The pan-C/EBP, Site2 of the AEP promoter, and their mutant oligodeoxynucleotide (ODN) decoys were synthetized as described in GeneDetect. HEK293 cells were transfected with these ODN decoys (final concentration is 2 µM) by using Lipofectamine 2000 (Invitrogen). Forty-eight hours post transfection, OGD treatment was done. Then EMSA assay using Site2 probe only was performed to detect the ability of C/EBPβ within the nuclear fragment binding to Site 2 of AEP promoter.

### Stereotaxic injection of virus in mouse hippocampus

The coding sequence of C/EBPβ, C/EBPα, HSF1 or shC/EBPβ, sh C/EBPα, shHSF1 were inserted into pFCGW-N1 lentiviral vectors (CMV promoter). The virus was packaged by viral vector core (VVC) of Emory Universtiy. Mice were anesthetized with isoflurane, and lentivirus (LV, 2 μl with similar titers >1 × 10^9^ IU/ml) were delivered at a rate of 0.3 μl/min (from Bregma, anteroposterior (AP) −2.2 mm, mediolateral (ML) ± 1.7 mm, dorsoventral (DV) −1.3 mm and −1.8 mm (two injections). Mice were assigned into gender- and age-matched treatment groups using a randomized block design.

### Aβ plaque histology

Amyloid plaques were stained with Thioflavin-S. Free-floating 40 μm brain sections were incubated in 0.25% potassium permanganate solution for 20 min, rinsed in distilled water, and incubated in bleaching solution containing 2% oxalic acid and 1% potassium metabisulfite for 2 min. After rinsed in distilled water, the sections were transferred to blocking solution containing 1% sodium hydroxide and 0.9% hydrogen peroxide for 20 min. The sections were incubated for 5 s in 0.25% acidic acid, then washed in distilled water and stained for 5 min with 0.0125% Thioflavin-S in 50% ethanol. The sections were washed with 50% ethanol and placed in distilled water. Then the sections were covered with a glass cover using mounting solution and examined under a fluorescence microscope. The plaque number and plaque area were calculated using the Image J software (National Institutes of Health). For immunohistochemical (IHC) visualization of Aβ, the brain sections were treated with 0.3% H_2_O_2_ for 10 min. Then, sections were washed three times in PBS and blocked in 1% BSA, 0.3% Triton X-100, for 30 min followed by overnight incubation with anti-Aβ antibody (1:500, Sigma-Aldrich) at 4 °C. The signal was developed using Histostain-SP kit.

### Golgi stain

Mouse brains were fixed in 10% formalin for 24 h, and then immersed in 3% potassium bichromate for 3 days in the dark. The solution was changed each day. Then the brains were transferred into 2% silver nitrate solution and incubated for 24 h in the dark. Vibratome sections were cut at 60 μm, air dried for 10 min, dehydrated through 95% and 100% ethanol, cleared in xylene and coverslipped.

### Electrophysiology

Acute hippocampal transversal slices were prepared from different ages of WT, 5XFAD, 3xTg mice injected with LV or AAV virus. Briefly, mice were anaesthetized with isoflurane, decapitated, and their brains dropped in ice-cold artificial cerebrospinal fluid (a-CSF) containing 124 mM NaCl, 3 mM KCl, 1.25 mM NaH_2_PO_4_, 6.0 mM MgCl_2_, 26 mM NaHCO_3_, 2.0 mM CaCl_2_, and 10 mM glucose. Hippocampi were dissected and cut into 400-μm thick transverse slices with a vibratome. After incubation at room temperature (23–24 °C) in a-CSF for 60–90 min, slices were placed in a recording chamber (RC-22C, Warner Instruments) on the stage of an up-right microscope (Olympus CX-31) and perfused at a rate of 3 ml per min with a-CSF (containing 1 mM MgCl_2_) at 23–24 °C. A 0.1 MΩ tungsten monopolar electrode was used to stimulate the Schaffer collaterals. The field excitatory post-synaptic potentials (fEPSPs) were recorded in CA1 stratum radiatum by a glass microelectrode filled with a-CSF with resistance of 3–4 MΩ. The stimulation output (Master-8; AMPI, Jerusalem) was controlled by the trigger function of an EPC9 amplifier (HEKA Elektronik, Lambrecht, Germany). fEPSPs were recorded under current-clamp mode. Data were filtered at 3 kHz and digitized at sampling rates of 20 kHz using Pulse software (HEKA Elektronik). The stimulus intensity (0.1 ms duration, 10–30 μA) was set to evoke 40% of the maximum f-EPSP and the test pulse was applied at a rate of 0.033 Hz. LTP of fEPSPs was induced by 3 theta-burst-stimulation (TBS), it is 4 pulses at 100 Hz, repeated three times with a 200-ms interval. The magnitudes of LTP are expressed as the mean percentage of baseline fEPSP initial slope.

### Morris water maze

Different ages of WT, 5XFAD, 3xTg mice injected with indicated LV or AAV virus were trained in a round, water-filled tub (52 inch diameter) in an environment rich with extra maze cues. An invisible escape platform was located in a fixed spatial location 1 cm below the water surface independent of a subject start position on a particular trial. In this manner, subjects needed to utilize extra maze cues to determine the platform’s location. At the beginning of each trial, the mouse was placed in the water maze with their paws touching the wall from one of four different starting positions (N, S, E, W). Each subject was given four trials/day for five consecutive days with a 15-min inter-trial interval. The maximum trial length was 60 s and if subjects did not reach the platform in the allotted time, they were manually guided to it. Upon reaching the invisible escape platform, subjects were left on it for an additional 5 s to allow for survey of the spatial cues in the environment to guide future navigation to the platform. After each trial, subjects were dried and kept in a dry plastic holding cage filled with paper towels to allow the subjects to dry off. The temperature of the water was monitored every hour so that mice were tested in water that was between 22 and 25 ° C. Following the 5 days of task acquisition, a probe trial was presented during which time the platform was removed and the percentage of time spent in the quadrant which previously contained the escape platform during task acquisition was measured over 60 s. All trials were analyzed for latency and swim speed by means of MazeScan (Clever Sys, Inc.).

### Statistical analysis

All data are expressed as mean ± s.e.m. from three or more independent experiments, and the level of significance between two groups was assessed with Student’s *t*-test. For more than two groups, one-way ANOVA followed by LSD post hoc test was applied. A value of *P* < 0.05 was considered to be statistically significant.

### Data availability

The data that support the findings of this study are available from the corresponding author upon reasonable request.

## Electronic supplementary material

Supplementary Information
