# Author Correction: C/EBPβ regulates delta-secretase expression and mediates pathogenesis in mouse models of Alzheimer’s disease

**DOI:** 10.1038/s41467-019-13553-z

**Published:** 2019-11-26

**Authors:** Zhi-Hao Wang, Ke Gong, Xia Liu, Zhentao Zhang, Xiaoou Sun, Zheng Zachory Wei, Shan Ping Yu, Fredric P. Manfredsson, Ivette M. Sandoval, Peter F. Johnson, Jianping Jia, Jian-Zhi Wang, Keqiang Ye

**Affiliations:** 1 Department of Pathology and Laboratory Medicine, Emory University School of Medicine, Atlanta, GA 30322 USA; 2 Department of Pathophysiology, Key Laboratory of Ministry of Education of Neurological Diseases, Tongji Medical College, Huazhong University of Science and Technology, 430030 Wuhan, China; 3 Department of Anesthesiology, Emory University School of Medicine, Atlanta, GA 30322 USA; 4 Department of Translational Science & Molecular Medicine, Michigan State University, 333 Bostwick Ave NE, Grand Rapids, MI 49503 USA; 5 Mouse Cancer Genetics Program, Center for Cancer Research, National Cancer Institute, Frederick, MD 21702 USA; 6 Department of Neurology, Xuanwu Hospital of Capital Medical University, 100053 Beijing, China; 7 Translational Center for Stem Cell Research, Tongji Hospital, Tongji University School of Medicine, 200065 Shanghai, China

**Keywords:** Transcription, Alzheimer's disease

Correction to: *Nature Communications* 10.1038/s41467-018-04120-z, published online 3 May 2018.

In the original version of the Article, the authors stated they had no competing interests to declare. They omitted to declare that Dr. Ye is a shareholder of Wuhan Yuanzheng Pharmaceuticals Inc. and Shanghai Braegen Pharmaceuticals Inc. This has now been corrected in the PDF and HTML versions of the article.

