# Supplementary material for: C/EBPβ regulates delta-secretase expression and mediates pathogenesis in mouse models of Alzheimer’s disease
Source: Nat Commun. 2018 May 3;9:1784. doi: 10.1038/s41467-018-04120-z (PMC5934399; doi:10.1038/s41467-018-04120-z)
Supplement: Supplementary file 1 — Supplementary Information [file 41467_2018_4120_MOESM1_ESM.pdf]

C/EBPβ regulates Delta-secretase expression and mediates pathogenesis in mouse models of Alzheimer's Disease

Wang et al.

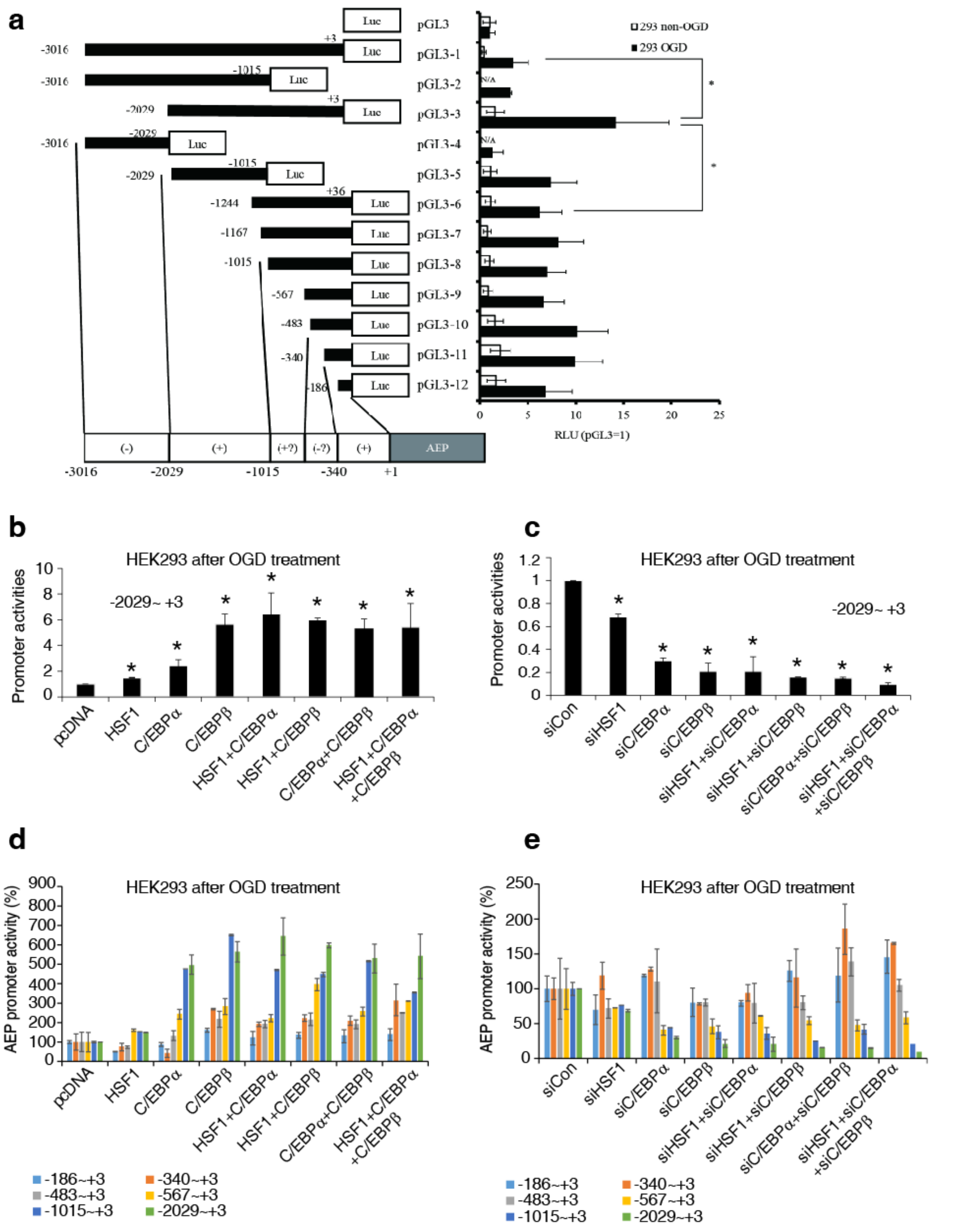

Supplementary Figure 1. The structure diagram of human LGMN promoter.

a, Different lengths of human LGMN promoter (+1: transcription start site) were constructed within a pGL3 luciferase reporter vector. These plasmids were transfected into HEK293 cells, followed by treatment with OGD/reperfusion. Luciferase activities were calculated by using Luciferase Reporter Assay System, which

indicated the promoter activities. The activity of pGL3 empty vector were normalized to one (mean ± s.e.m.; *P<0.05, one-way ANOVA). **b&c,** LMGN promoter activity assay. The pGL3 luciferase reporter plasmids (-2029~+3 of AEP) were co-transfected with HSF1/CEBPα/CEBPβ overexpression plasmids (b) or their siRNA (c) into HEK293 cells. The luciferase activities were measured. The experiments were repeated three times, and duplicated samples were tested each time (mean ± s.e.m.; *P<0.05, one-way ANOVA). **d&e,** Mapping the promoter activity. Different lengths of human LGMN promoter plasmids were transfected into HEK293 cells, in the presence of HSF1 or CEBPα or CEBPβ plasmids (**d**) or their respective siRNA (**e**). Luciferase assay was performed to detect these promoter activities. Experiments were repeated at least 3 times.

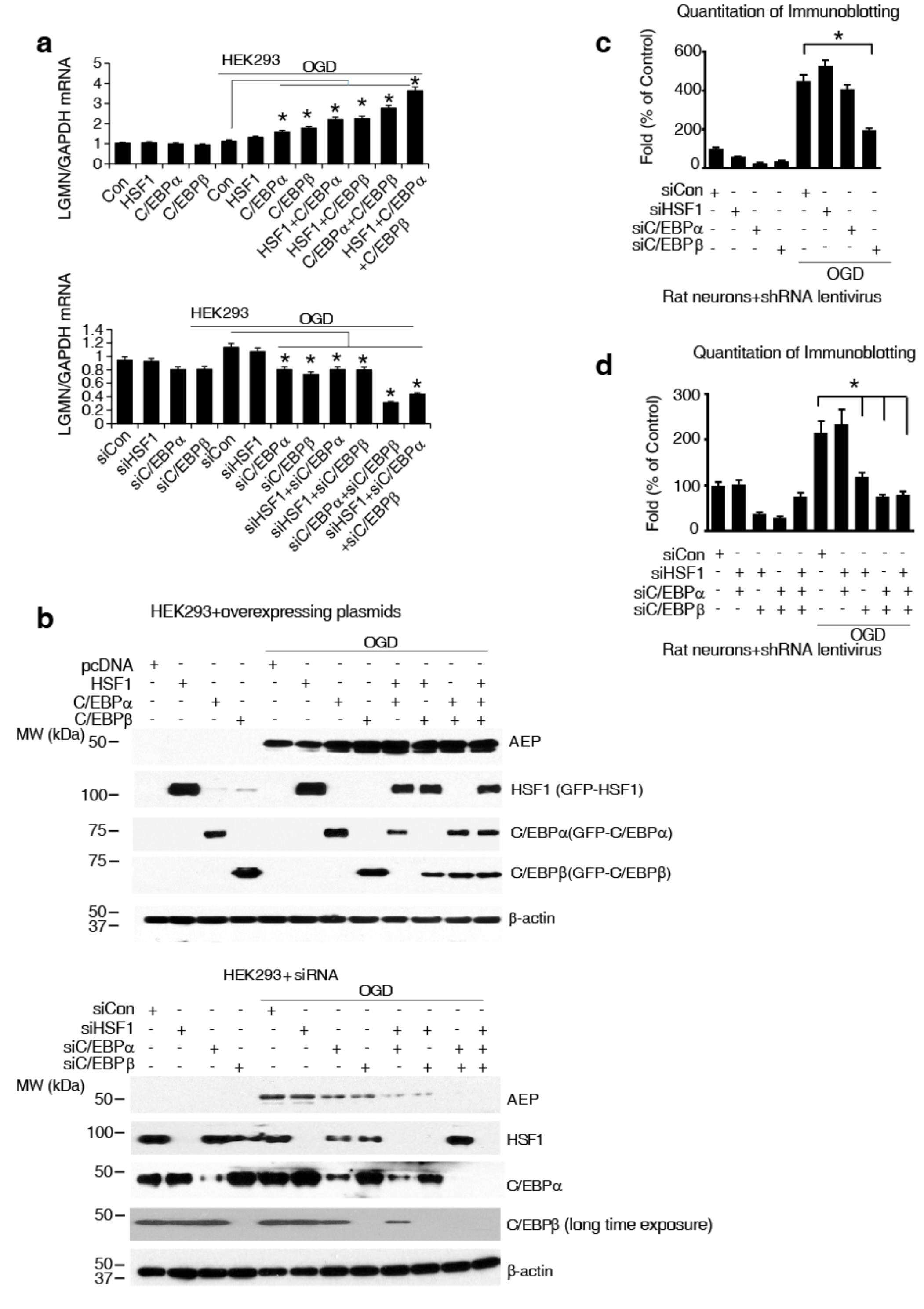

Supplementary Figure 2. C/EBPα and C/EBPβ regulate delta-secretase in HEK293 cells by OGD.

a&b, C/EBPs mediate delta-secretase expression in HEK293 cells. HEK293 cells

were seeded in 6-well plates and transfected with overexpression plasmids or siRNAs against HSF1, C/EBPα, and C/EBPβ. After 48 h, OGD/reperfusion treatment was performed on these cells. Then the cell lysates were analyzed by real-time PCR (a) and western blotting (b) for detecting the mRNA and protein levels of AEP. HSF1, C/EBPα, and C/EBPβ's protein levels were detected by western blotting as well (b). Data are representative of three independent experiments (mean ± s.e.m.; *P<0.05, one-way ANOVA). **c&d,** Quantitative description of the western blot results (relative to control gene) in Figure 1f (mean ± s.e.m.; *P<0.05, one-way ANOVA).

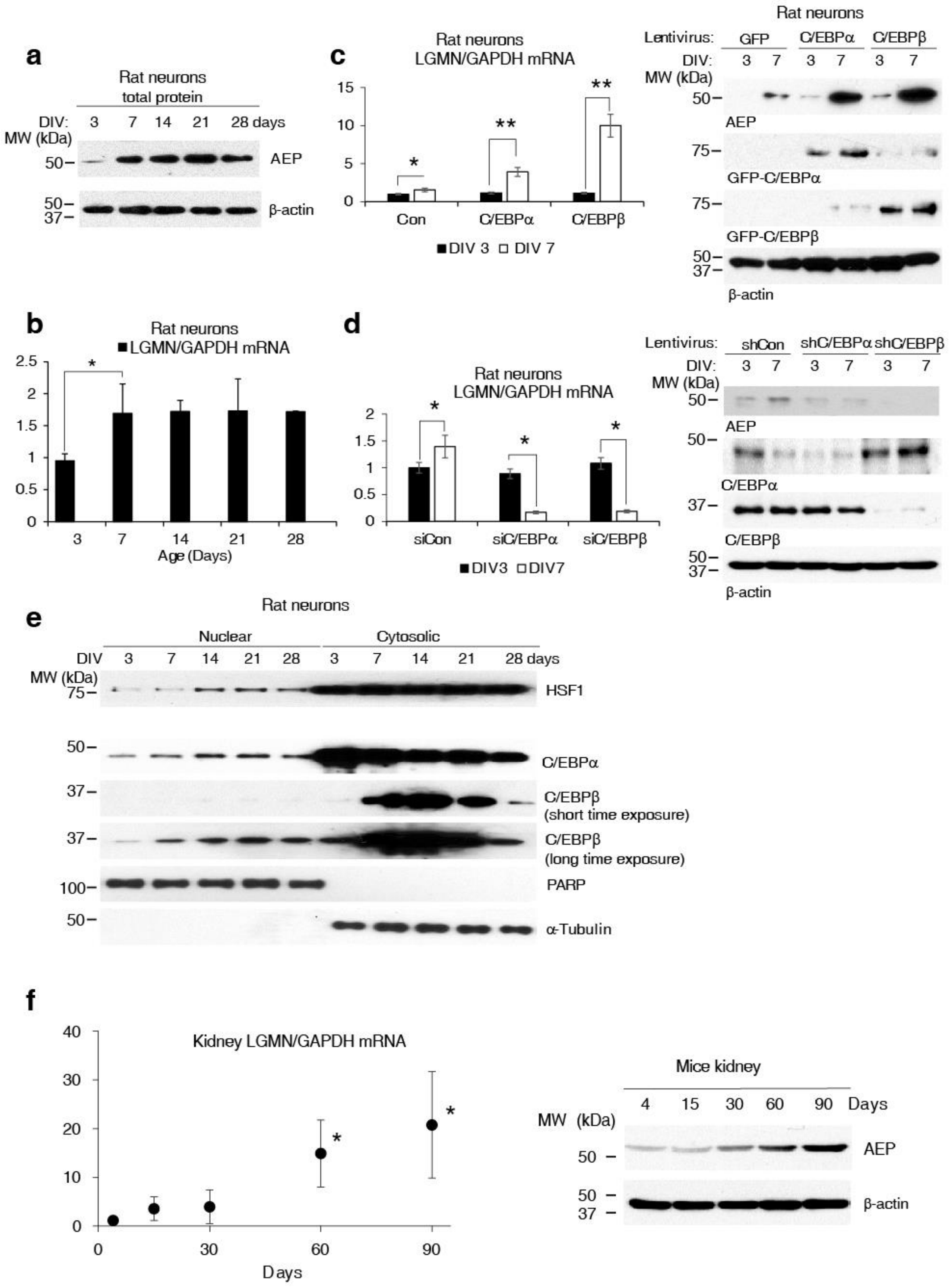

Supplementary Figure 3. C/EBPβ increases delta-secretase in primary neuronal cultures during maturation.

a&b, Delta-secretase expression is elevated during neuronal maturation. Primary cultures of neurons from rat E17 embryos were seeded in 6-well plates. At DIV 3, 7,

14, 21, and 28, neurons were subjected to western blotting (a) and real-time PCR analysis (b) for detecting the protein and mRNA levels of delta-secretase. **c&d,** Primary cultures of neurons from P0 newborn rats were transduced with C/EBPα, β, and control (GFP) lentivirus (c), or shC/EBPα, β, and shCon lentivirus (d) at DIV1 or DIV3. Neurons were harvested at DIV3 (infection for 2 days) or DIV7 (infection for 4 days). AEP mRNAs and protein levels were analyzed by qRT-PCR (left) and immunoblotting (right), respectively. **e,** C/EBPβ is expressed in the nucleus in a time-dependent manner. The nuclear and cytosolic fractions were isolated from primary neuronal cultures at indicated DIV. Western blotting analysis were performed for detecting C/EBPα and C/EBPβ expression at each fraction. PARP and α-tubulin's levels were employed as loading controls of nuclear and cytosolic proteins, respectively. Western blot data in **a**, **c**, **d** and **e** are representative of three independent experiments. Real-time PCR data in **b**, **c** and **d** represent mean ± s.e.m. of three independent experiments. (*P < 0.05, **P < 0.01, Student's t-test). **f,** Quantitative RT-PCR analysis of delta-secretase from different ages of C57/BL6J mice (left panel) (mean ± s.e.m.; n = 4; *P < 0.05, one-way ANOVA). Delta-secretase levels increase in an age-dependent manner. Immunoblotting analysis of kidney lysates from different ages of wild-type mice with anti-AEP (right panels). Western blots are representative of three independent experiments.

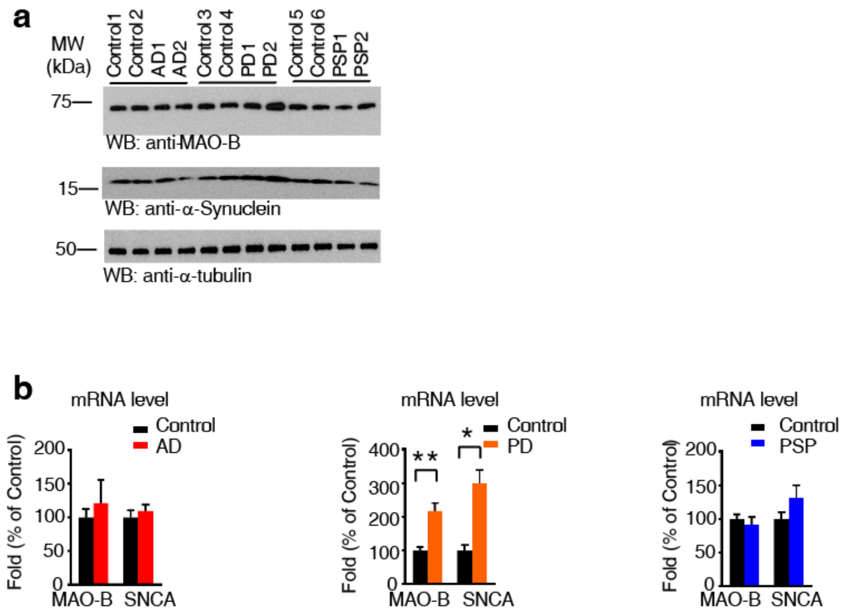

Supplementary Figure 4. The downstream targets of C/EBPβ are selectively expressed in neurodegenerative diseases.

a&b, Protein (a) and mRNA (b) levels of α-Synuclein and MAO-B, two reported downstream targets of C/EBPbeta in AD, PD and PSP patient brains. α-Synuclein and MAO-B were not altered in AD as detected by immunoblotting (a) and real-time PCR (b, mean ± s.e.m.; n = 4; *P < 0.05, **P < 0.01, one-way ANOVA). Western blots are representative of three independent experiments.

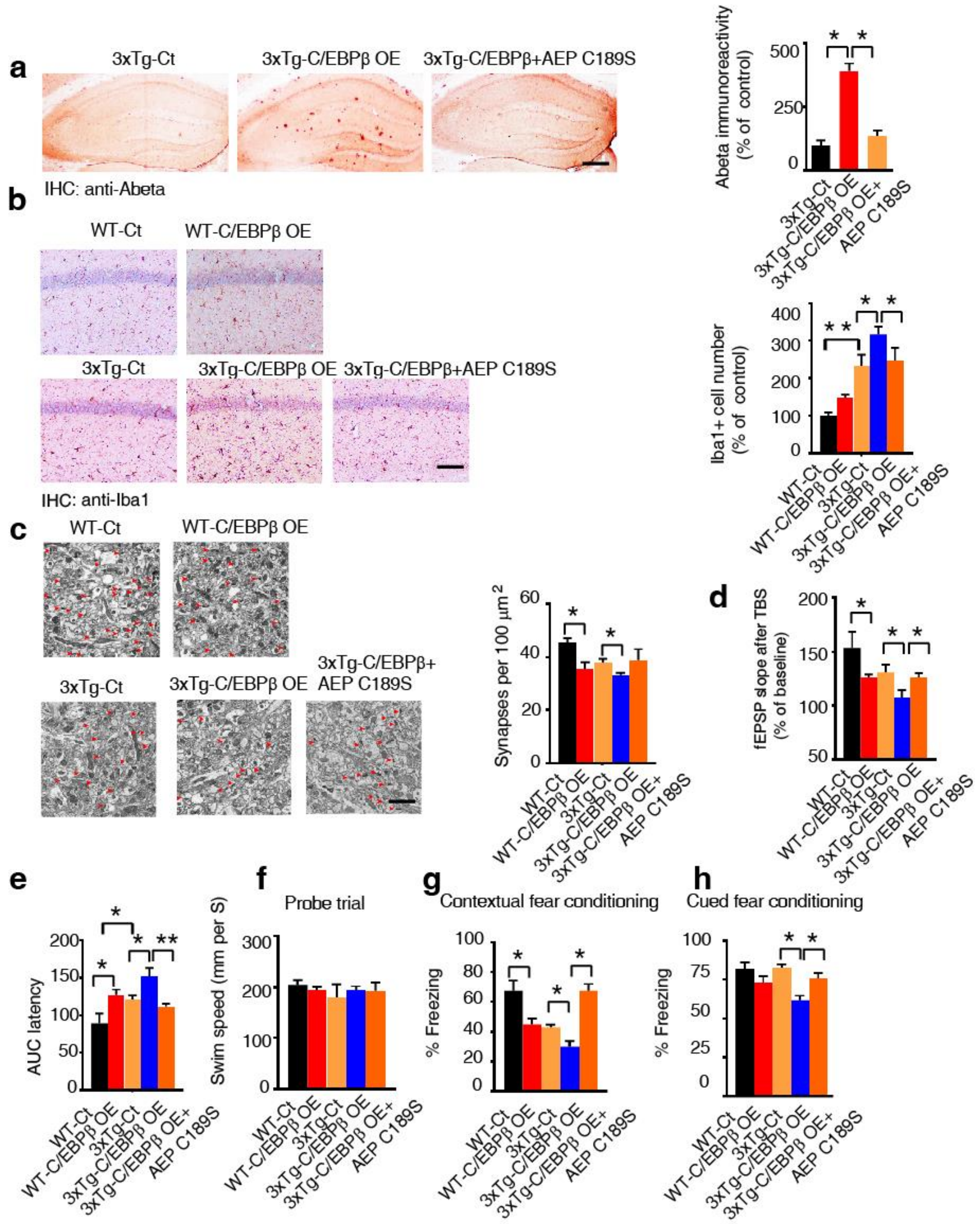

Supplementary Figure 5. **Overexpression of C/EBPβ in young 3xTg mice accelerates AD-like pathogenesis, exacerbating cognitive impairments.**

a, Overexpression of C/EBPβ increases amyloid plaques in hippocampus of 3xTg AD mice. IHC staining of Aβ in the hippocampus (left panels, scale bar, 300 μm). Quantitative analysis of Aβ immunoreactivity (right panel). Data represent mean ± s.e.m. of 12–18 sections from 3 mice in each group (*P < 0.05, one-way ANOVA). **b,**

Overexpression of C/EBPβ enhances neuro-inflammation. Iba1 IHC staining was elevated in 3xTg/C/EBPβ mice as compared to 3xTg/control mice. Expression of C189S repressed the increased Iba-1 signaling by C/EBPβ (bottom left panel, scale bar, 50 μm). Quantitative analysis of Iba1 positive cells (right panel). Data represent mean ± s.e.m. of 11–15 sections from 3 mice in each group (*P < 0.05, **P < 0.01, one-way ANOVA). **c,** C/EBPβ overexpression decreases the synapses in the hippocampus. Electron microscopy analysis of synapses. C189S reversed the synapse loss elicited by C/EBPβ in 3xTg mice (left panels, n = 6 mice per group). Scale bar, 1 μm. Quantitative analysis of synapses in hippocampus (right panels, mean ± s.e.m.; n = 6 mice per group; *P < 0.05, one-way ANOVA). **d,** fEPSP slope analysis. Overexpression of C/EBPβ reduced the fEPSP slopes in wild-type and 3xTg mice, respectively. C189S mutant reduced the inhibitory effect by C/EBPβ in 3xTg mice (n=6, *P < 0.05, one-way ANOVA). **e,** Integrated latency (AUC) for mice injected with virus (mean ± s.e.m.; n = 7-8 mice per group; *P < 0.05, **P < 0.01, one-way ANOVA). **f,** The swim speed of the tested groups was the same in Morris Water Maze. (mean ± s.e.m.; n = 7-8 mice per group; P>0.05, one-way ANOVA). **g&h**, Fear condition tests. Contextual and cued fear conditioning was reduced in C/EBPβ overexpressed mice, which were reversed by C189S mutant in 3xTg AD mice (mean ± s.e.m.; n = 7-8 mice per group; *P < 0.05, one-way ANOVA).

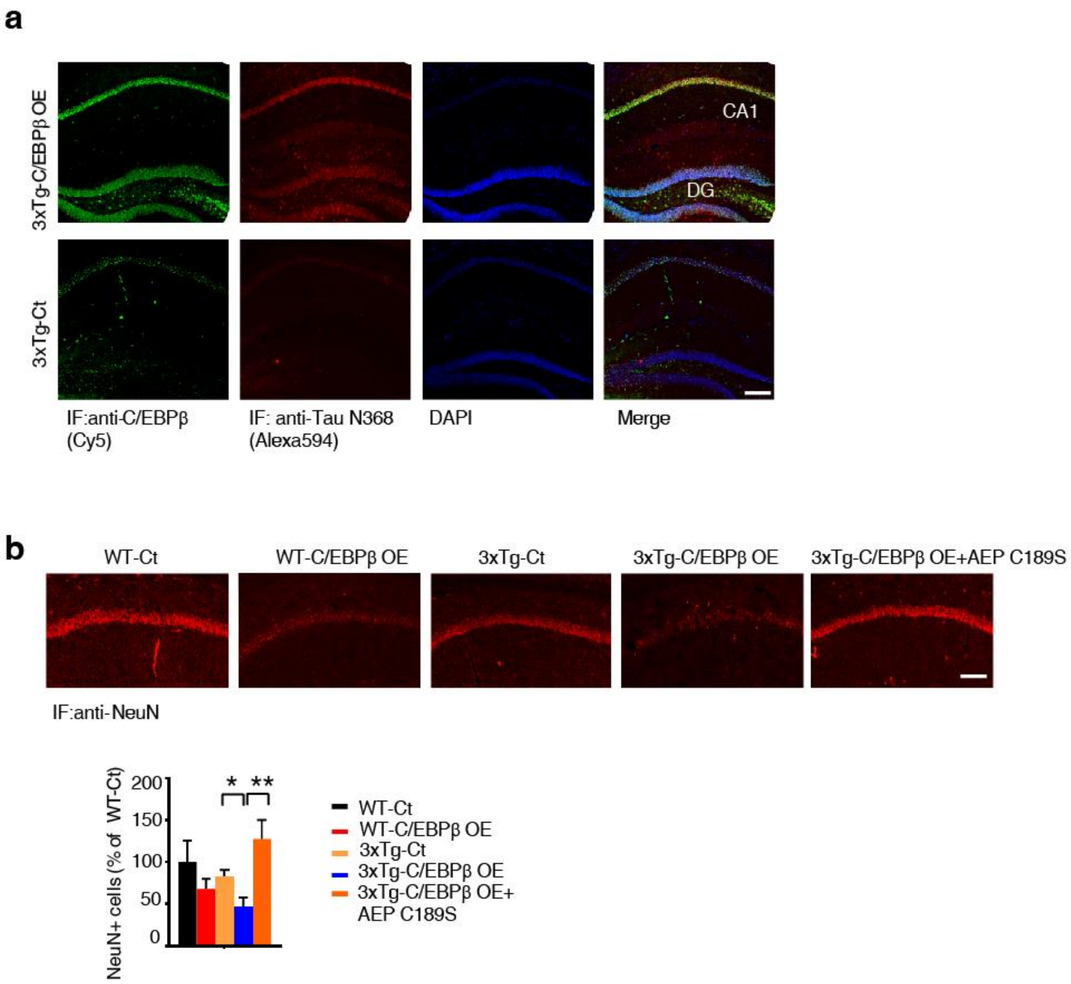

Supplementary Figure 6. **Overexpression of C/EBPβ in 3xTg mice accelerates Tau cleavage and neuronal loss.**

a, C/EBPβ and Tau N368 staining in hippocampal regions. Overexpression of C/EBPβ significantly escalated Tau cleavage by AEP in 3xTg mice. Data represent mean ± s.e.m. of 12–18 sections from 3 mice in each group (*$P < 0.05$, one-way ANOVA). Scale bar, 150 µm. **b,** NeuN immunostaining. C/EBPβ overexpression triggered neuronal loss in 3xTg mice, whereas dominant-negative AEP C189S mutant blocked this effect. Data represent mean ± s.e.m. of 10–18 sections from 3 mice in each group (*$P < 0.05$, **$P < 0.01$, one-way ANOVA). Scale bar, 150 µm.

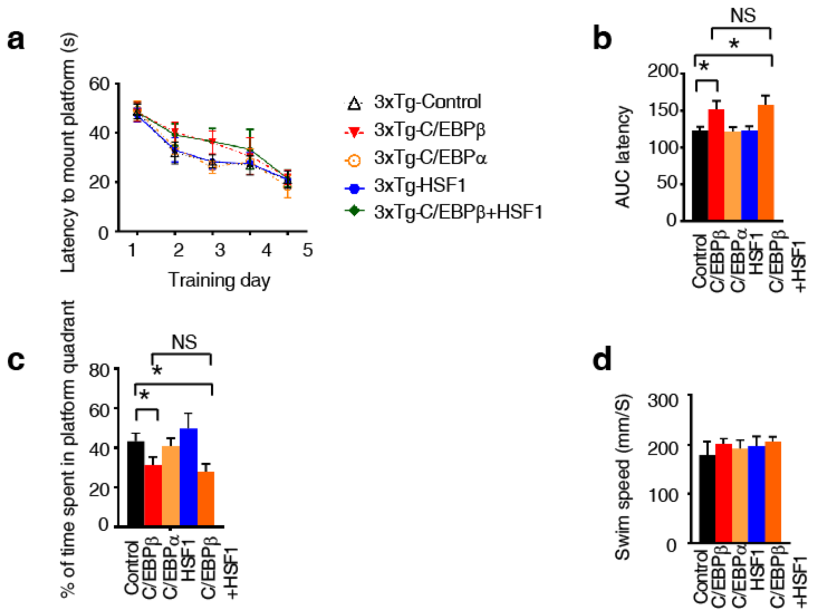

Supplementary Figure 7. Overexpression of C/EBPα or HSF1 in young 3xTg mice does not worsen cognitive dysfunctions.

a-c, Morris Water Maze analysis of cognitive functions. C/EBPα or HSF1 overexpression in the hippocampus did not exacerbate the learning and memory dysfunctions in young 3xTg mice (mean ±s.e.m.; n = 7-8 mice per group; *P< 0.05, one-way ANOVA). **b,** Integrated latency (AUC) for transgenic mice (mean ±s.e.m.; n = 7-8 mice per group; *P < 0.05, Student's t-test). **d,** The swim speed of in MWM test remain comparable (mean ±s.e.m.; n = 7-8 mice per group).

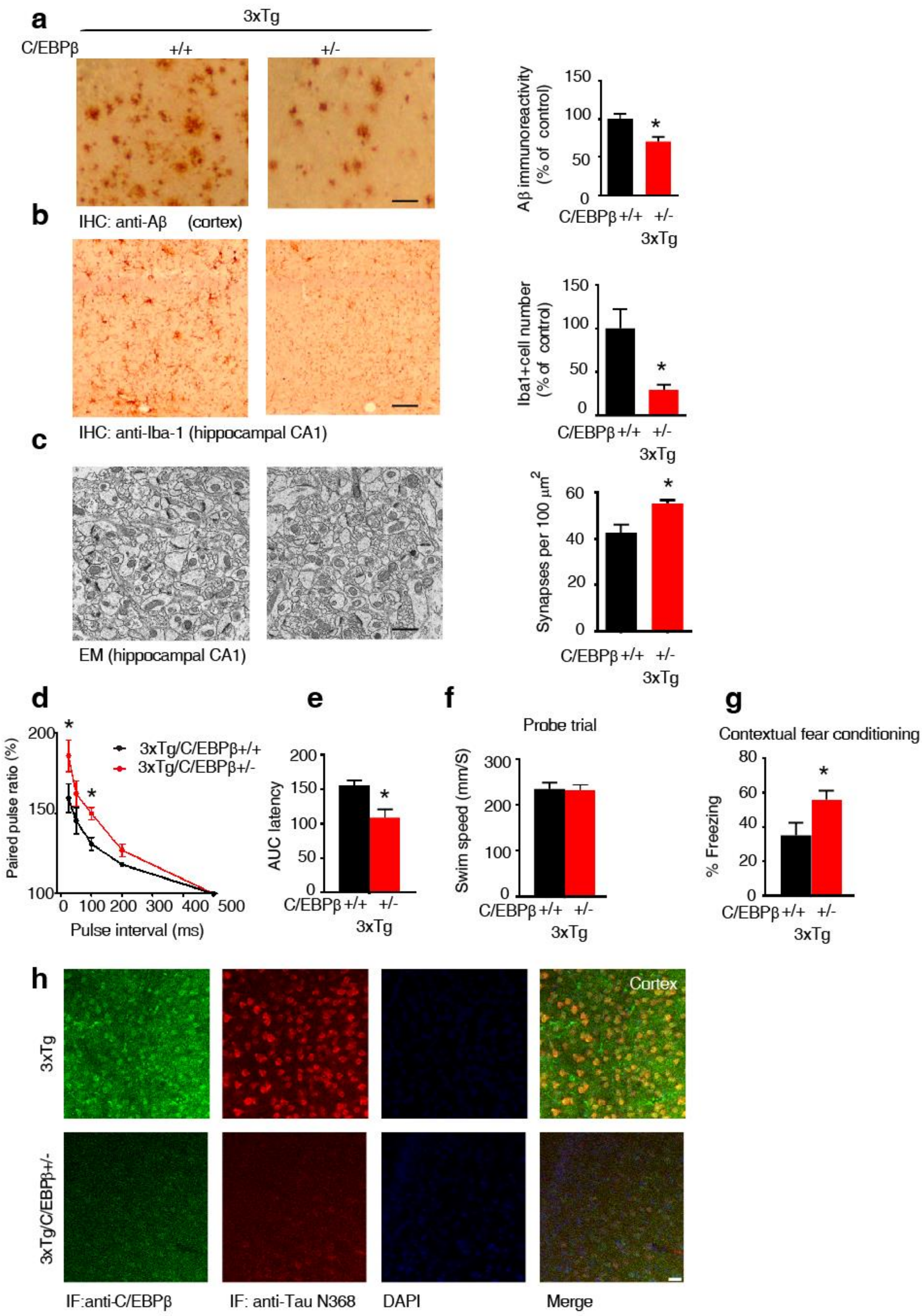

Supplementary Figure 8. **Reduction of C/EBPβ from 3xTg mice reduces delta-secretase expression and AD-like pathogenesis, rescuing cognitive functions.**

a, Amyloid plaques are reduced in 3xTg mice following C/EBPβ knockdown. IHC staining with anti-Aβ antibody on the cortex from the brain sections of 10 month old 3xTg/C/EBPβ +/- and 3xTg mice (left panels, scale bar, 50 μm). Quantification of Aβ immunoreactivity (right panel, mean ± s.e.m. of 11–18 sections from 3 mice in each group; *P < 0.05, Student's t-test). **b,** Microglial activation is decreased in 3xTg mice, when C/EBPβ is silenced. IHC staining with anti-Iba-1 on the hippocampus from brain sections of 10 month old 3xTg/C/EBPβ +/- and 3xTg mice (left panels, scale bar, 50 μm). Quantification of Iba-1 positive cells (right panel, mean ± s.e.m. of 13–18 sections from 3 mice in each group; *P < 0.05, Student's t-test). **c,** The synapses from the hippocampus of 10 month old 3xTg/C/EBPβ +/- and 3xTg mice were determined by electron microscopy (mean ± s.e.m.; n = 6; *P < 0.05, Student's t-test). Scale bar, 1 μm. **d,** The ratio of paired pulses (mean ± s.e.m.; n = 6 in each group; *P < 0.05, Student's t-test). **e,** Integrated latency (AUC) for transgenic mice (mean ± s.e.m.; n = 8 mice per group; *P < 0.05, Student's t-test)**. f,** The swim speed of 3xTg/C/EBPβ +/- and 3xTg mice in MWM test remained comparable (mean ± s.e.m.; n = 8 mice per group)**. g,** Fear condition tests. Contextual fear conditioning was reduced in 3xTg mice, which were reversed in 3xTg/C/EBPβ +/- mice (mean ± s.e.m.; n = 8 mice per group; *P<0.05, Student's t-test)**. h,** C/EBPβ and AEP-cleaved Tau N368 were highly escalated in 10 months old 3xTg mice cortex as compared with age-matched 3xTg/C/EBPβ +/- mice. Scale bar, 20 μm.

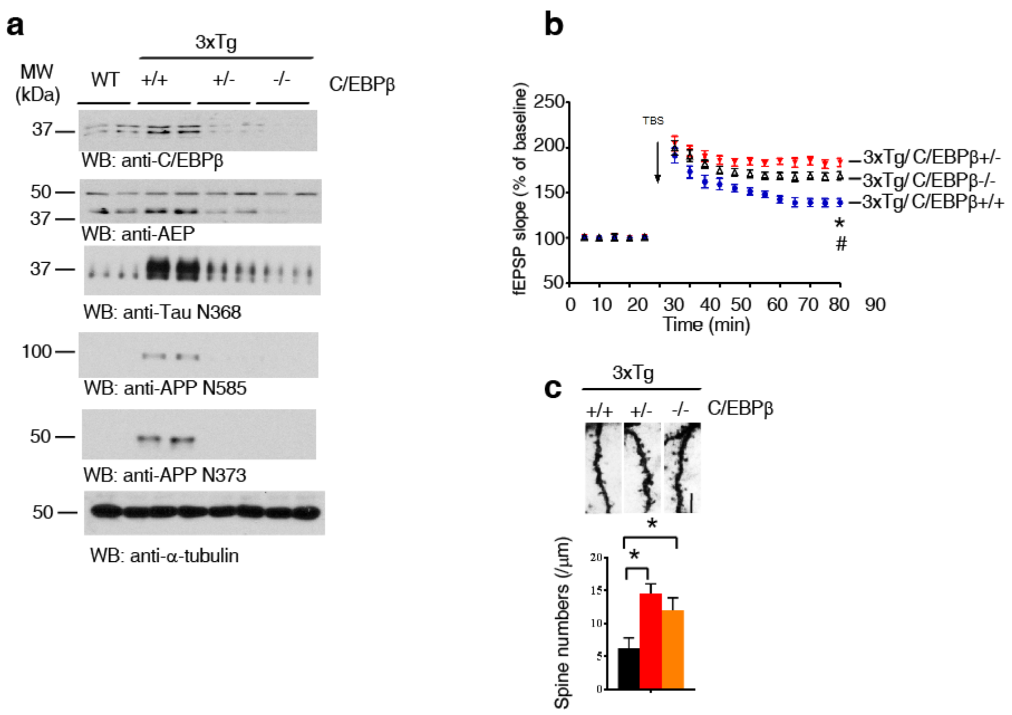

Supplementary Figure 9. Knockout of C/EBPβ from 3xTg mice reduces delta-secretase expression and rescues impairment of synaptic plasticity.

a, Knockout of C/EBPβ decreases delta-secretase and APP and Tau cleavage by delta-secretase. The hippocampal tissues from both 10 months old 3xTg, 3xTg/C/EBPβ +/– and 3xTg/C/EBPβ –/– mice were analyzed by immunoblotting with various antibodies (n = 2 mice per group). **b,** Electrophysiology analysis. C/EBPβ knockout in the hippocampus rescued the LTP defects in aged 3xTg mice (mean ± s.e.m.; n = 6 in each group; *P < 0.05 3xTg/C/EBPβ +/+ compared with 3xTg/C/EBPβ +/–, # P < 0.05 3xTg/C/EBPβ +/+ compared with 3xTg/C/EBPβ –/–, one-way ANOVA). **c,** Spine density in the hippocampus determined by Golgi staining. Golgi staining was conducted on brain sections from 10 month old 3xTg, 3xTg/C/EBPβ +/– and 3xTg/C/EBPβ –/– mice hippocampal regions. (mean ± s.e.m.; n = 4; *p < 0.05, Student's t-test). Scale bar, 5 μm.

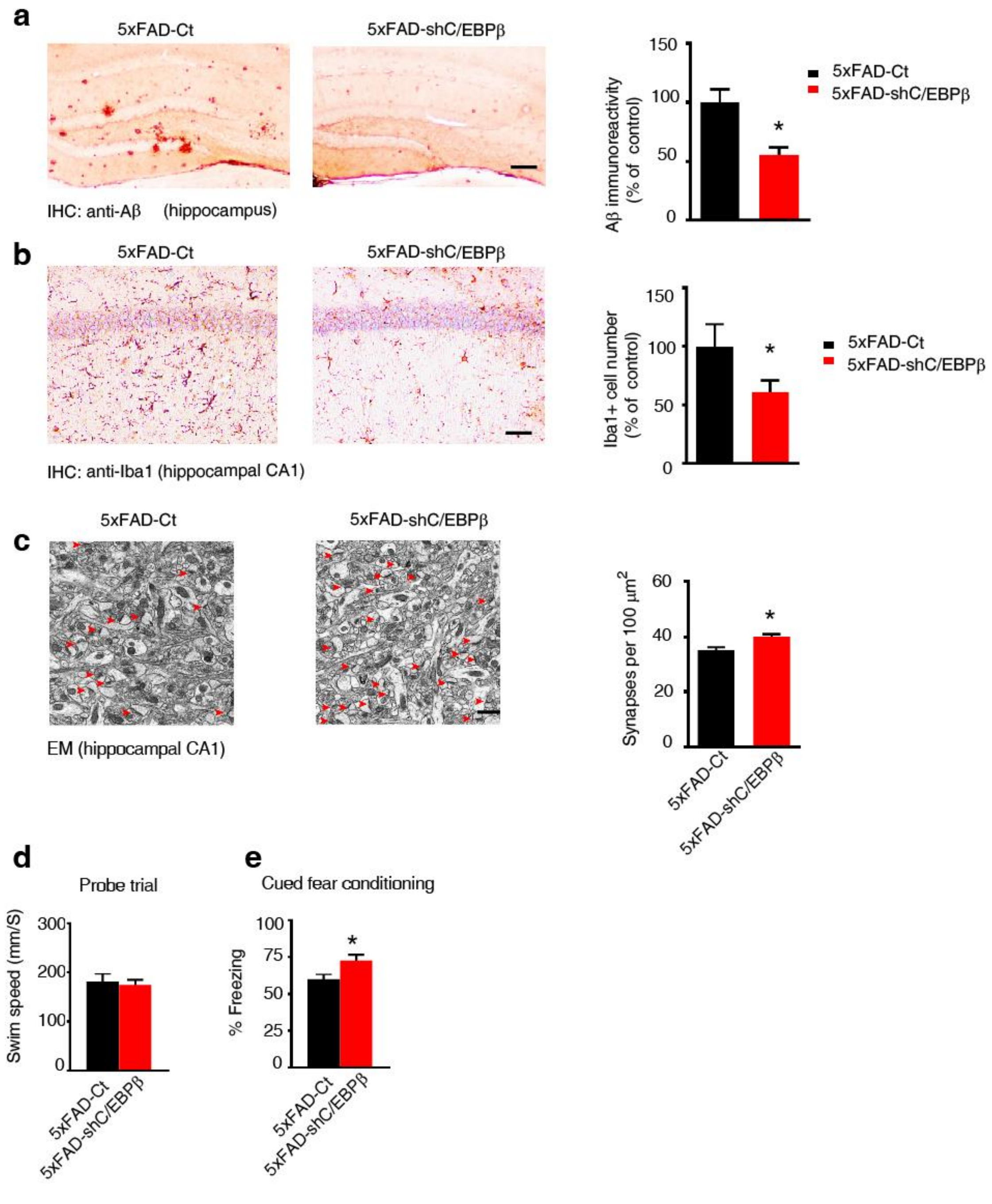

Supplementary Figure 10. **Knockdown of C/EBPβ from 5XFAD mice reduces delta-secretase and AD-like pathogenesis, improving cognitive functions.**

a, Depletion of C/EBPβ reduces amyloid plaques. Amyloid plaques were analyzed by IHC staining with anti-Aβ (left panels, scale bar, 100 μm). Quantification of Aβ immuno-reactivity (right panel, mean ± s.e.m. of 11–16 sections from 3 mice in each

group; *P < 0.05, Student's t-test). **b,** Depletion of C/EBPβ reduces neuro-inflammation. Iba-1 IHC staining was reduced in C/EBPβ-depleted 5XFAD mice (left panel, scale bar, 50 μm). Quantification of Iba-1 positive cells (right panel, mean ± s.e.m. of 16–18 sections from 3 mice in each group; NS represents no significance， *P < 0.05, Student's t-test). **c,** C/EBPβ depletion increases synapses in the hippocampus. Electron microscopy analysis of brain sections from C/EBPβ-depleted 5XFAD mice. (mean ± s.e.m.; n = 6; NS represents no significance， *P < 0.05, Student's t-test). Scale bar, 1 μm. **d,** The swim speed of both groups is the same in Morris Water Maze (mean ± s.e.m.; n = 8 mice per group)**. e,** Fear condition tests. The cued fear conditioning was enhanced when C/EBPβ was depleted in 5XFAD mice (mean ± s.e.m.; n =8 mice per group; *P<0.05, Student's t-test)**.**

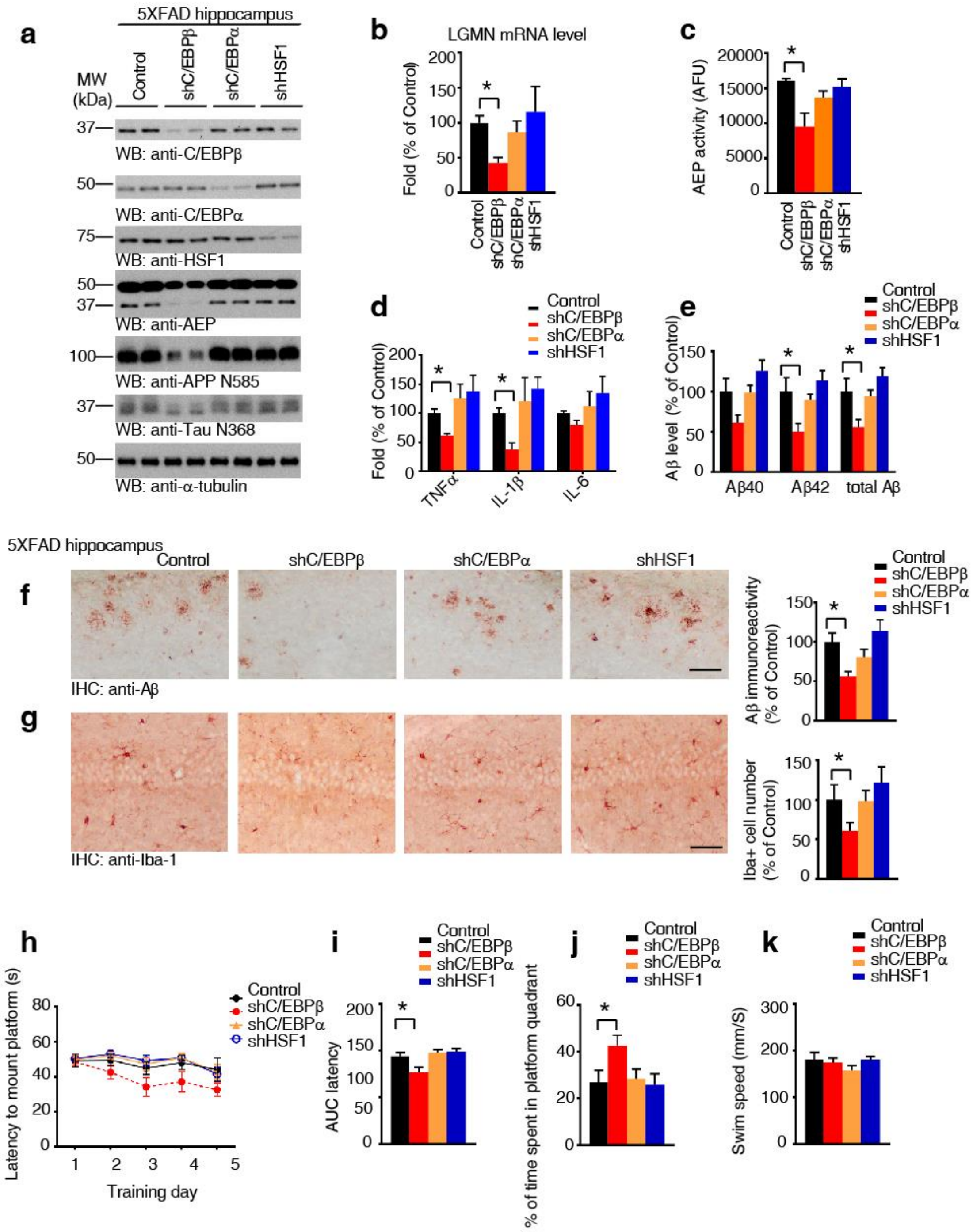

Supplementary Figure 11. Knockdown of C/EBPα or HSF1 in 5XFAD mice does not reduce AD-like pathogenesis and cognitive function.

a, Knockdown of C/EBPα or HSF1 does not reduce delta-secretase and APP and Tau fragments cleaved by delta-secretase. Hippocampal tissues from both control and LV–shRNA injected 5XFAD mice were analyzed by immunoblotting with various

antibodies (n = 3 mice per group). **b&c**, Knockdown of C/EBPα or HSF1 does not reduce delta-secretase mRNA and enzymatic activities. Data represent mean ± s.e.m. of 3 mice per group (*$P < 0.05$, Student's t-test). **d,** Knockdown of C/EBPα or HSF1 does not reduce expression levels of inflammatory cytokines. Data represent mean ± s.e.m. of 3 mice per group (*$P < 0.05$, Student's t-test). **e,** C/EBPα or HSF1 knockdown does not decrease Aβ level in 5XFAD mice. Aβ ELISA represents mean ± s.e.m. of 3 mice per group (*$P < 0.05$, Student's t-test). **f-g,** IHC staining of Aβ and Iba-1 in hippocampal CA1. Data represent mean ± s.e.m. of 12–18 sections from 3 mice in each group (*$P < 0.05$, one-way ANOVA). Scale bar, 50 μm. **h-j,** Morris Water Maze analysis of cognitive functions. C/EBPα or HSF1 redution in the hippocampus did not rescue the learning and memory dysfunctions in 5XFAD mice (mean ± s.e.m.; n = 7-8 mice per group; *$P< 0.05$, one-way ANOVA). **i,** Integrated latency (AUC) for transgenic mice (mean ± s.e.m.; n = 7-8 mice per group; *$P < 0.05$, Student's t-test). **k,** The swim speed of in Morris Water Maze test remain comparable (mean ± s.e.m.; n = 7-8 mice per group).

Figure 1

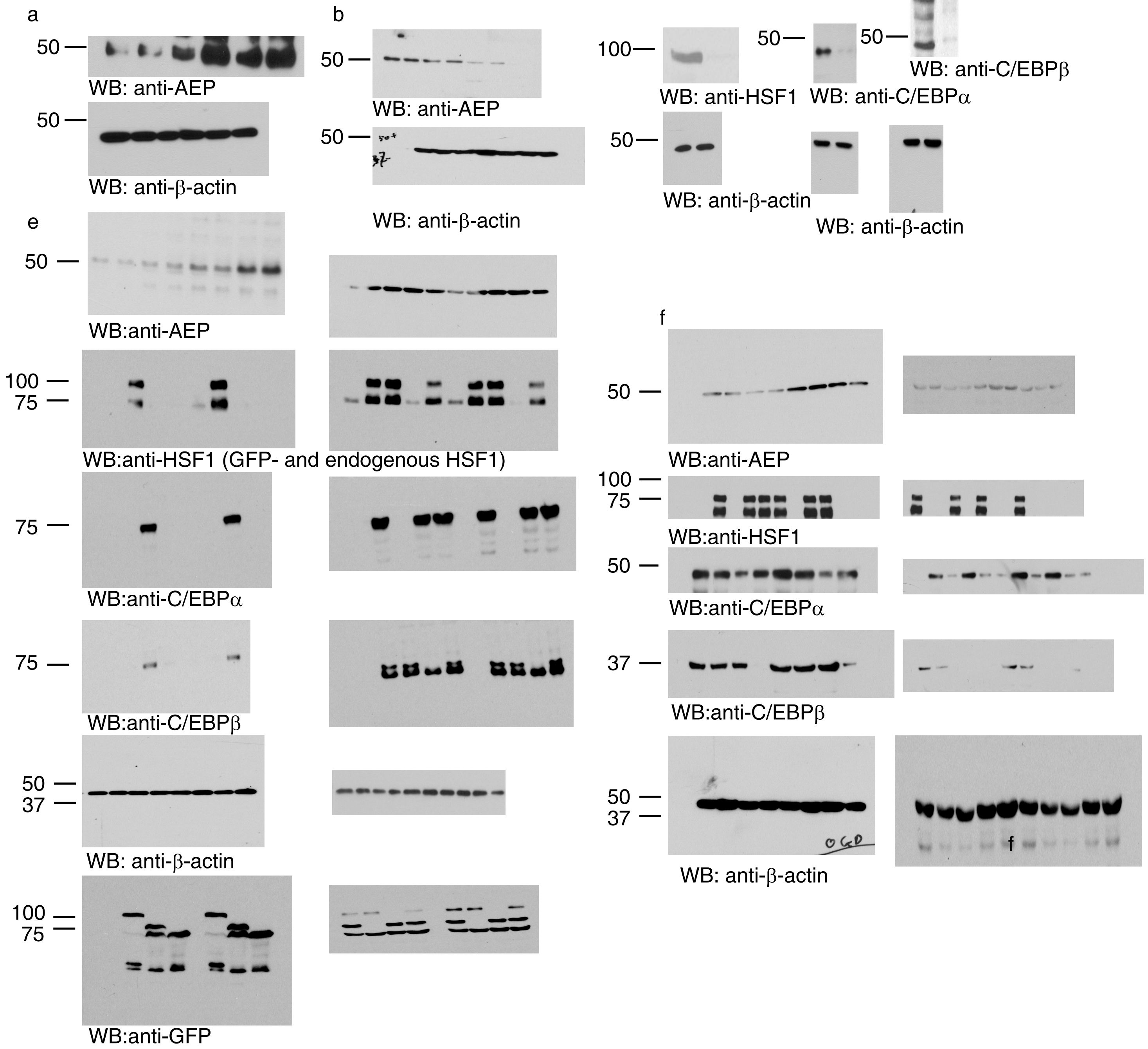

Figure 2

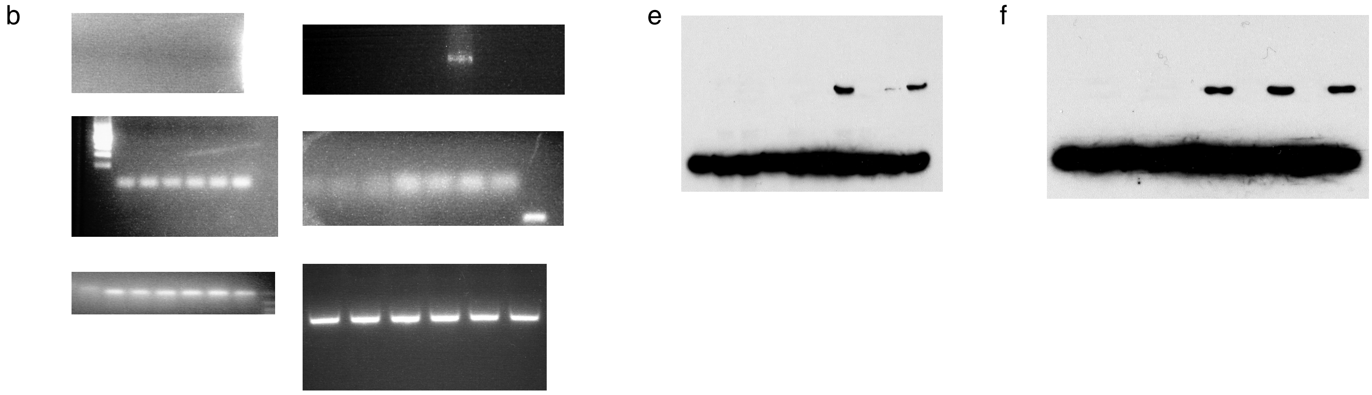

Supplementary Figure 12. Uncropped blots and gels data for Figures

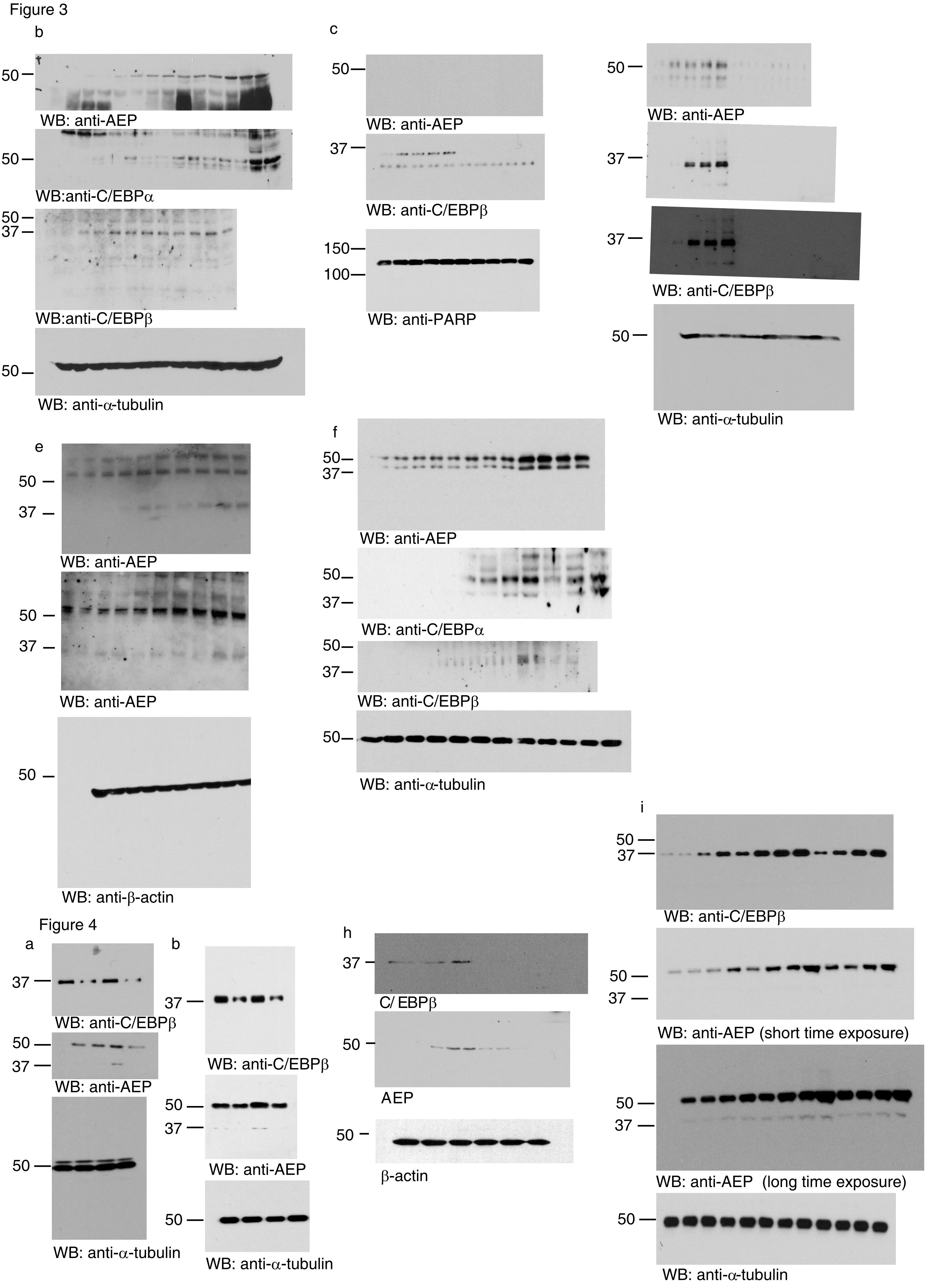

Supplementary Figure 13. Uncropped blots and gels data for Figures

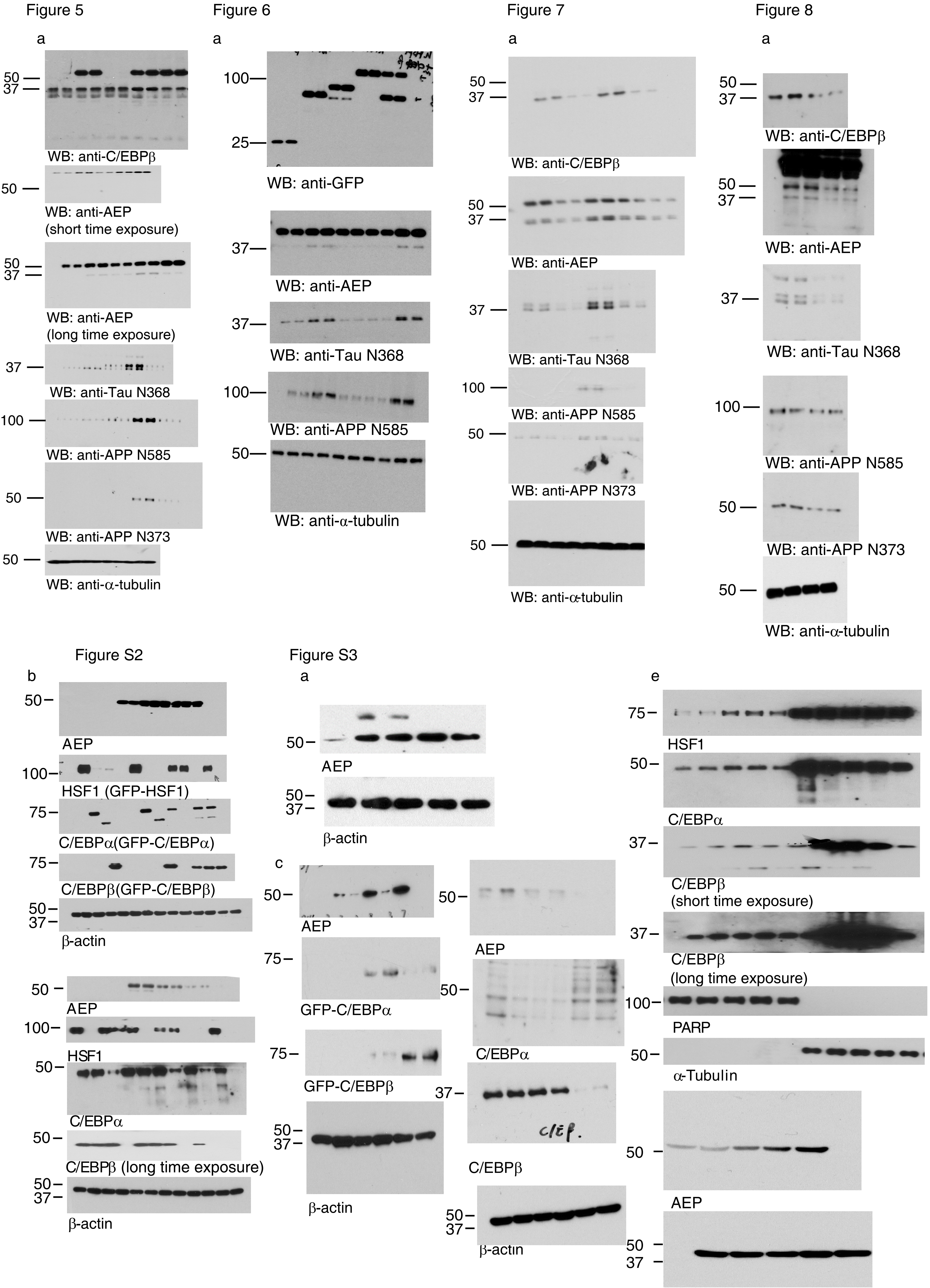

Supplementary Figure 14. Uncropped blots and gels data for Figures

Figure S4

a

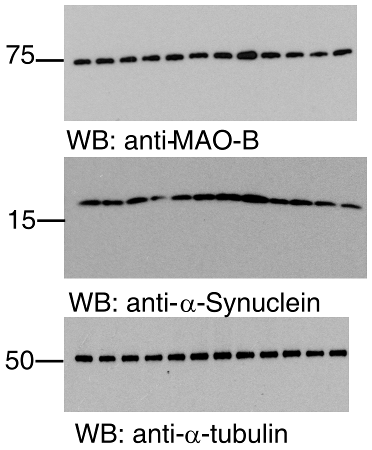

Figure S9

a

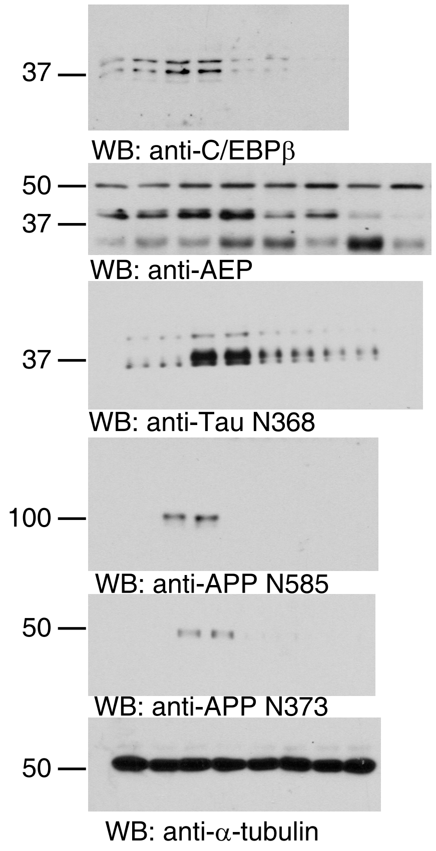

Figure S11

a

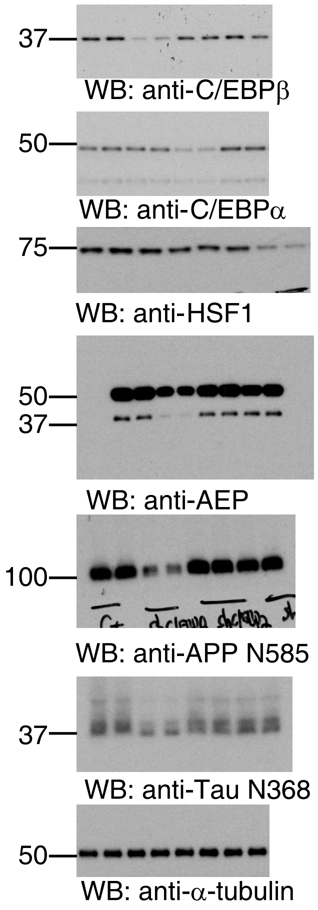

Supplementary Figure 15. Uncropped blots and gels data for Figures

Mice line	Virus injection	Behavior test	Other experiments
3xTg	3-month-old	6-month-old	6.5-month-old
WT	3-month-old	6-month-old	6.5-month-old
WT/C/EBPβ +/- or -/-	None	10-month-old	11-month-old
3xTg/C/EBPβ +/- or -/-	None	10-month-old	11-month-old
5XFAD	2.5-month-old	5.5-month-old	6-month-old

Supplementary Table 1. The age of AD mouse models used in animal experiments

The detailed age information of different AD mouse models in animal experiments is summarized**.**
